# Cancer nanomedicine and RNA/Lipid nanoparticle platforms in oncology: developmental evolution, translational barriers, and precision-therapy opportunities

**DOI:** 10.1186/s43046-026-00418-y

**Published:** 2026-10-16

**Authors:** Bhasker sambar, Anil Kumar Dudam

**Affiliations:** 1 R&D, Alvogen Inc, Pine Brook, USA; 2 Regulatory Affairs CMC, STA Pharmaceuticals US LLC, Sandiego, USA

**Keywords:** Cancer nanomedicine, Lipid nanoparticles, Liposomes, Albumin-bound nanoparticles, mRNA cancer vaccines, siRNA, Tumor microenvironment, Precision oncology, Critical quality attributes, Translational oncology

## Abstract

Nanomedicine has become an important translational platform in oncology by enabling altered pharmacokinetics, controlled exposure, reduced systemic toxicity, intracellular delivery of nucleic acids, and improved handling of complex therapeutic payloads. Early cancer nanomedicines, including pegylated liposomal cytotoxics and albumin-bound drug products, demonstrated that nanoscale formulation design could improve the clinical utility of established anticancer agents. More recently, lipid nanoparticles, RNA therapeutics, individualized neoantigen vaccines, gene-regulatory approaches, and immune-modulating delivery systems have expanded the role of nanoparticles from passive drug carriers to programmable oncology platforms. This narrative review examines the developmental evolution of cancer-focused pharmaceutical nanoparticles, with emphasis on liposomes, lipid nanoparticles (LNPs), albumin-bound systems, polymeric particles, polymeric micelles, nanocrystals, nanoemulsions, solid lipid nanoparticles, inorganic particles, extracellular vesicles, and hybrid delivery technologies. Each platform class is treated systematically, covering representative approved products, formulation characteristics, route of administration, clinical evidence, and the specific development risks that limit it. There view connects platform design with oncology-specific barriers, including tumor heterogeneity, variable enhanced permeability and retention (EPR) effects, dense extracellular matrix, abnormal tumor vasculature, mononuclear phagocyte clearance, protein-corona formation, endosomal escape, immune activation, and the difficulty of translating animal tumor models into clinical benefit. These barriers are organized as a sequential delivery cascade, and the behavior of each platform class is compared step by step rather than described as a set of universal phenomena. Nanoparticle candidates that accumulated well in preclinical tumors but failed in clinical trials are examined alongside approved products, because the contrast between the two is where most of the transferable lessons sit. A product-development perspective is used to discuss critical quality attributes (CQAs), manufacturing scalability, potency testing, sterility strategy, stability, release methods, regulatory expectations, commercial feasibility, and clinical differentiation. A development-to-translation framework is proposed for comparing cancer nanomedicine platforms across formulation maturity, analytical maturity, manufacturing maturity, biological rationale, clinical evidence, regulatory readiness, and commercial feasibility; regulatory and commercial maturity are scored separately, the criteria behind each level are defined explicitly, and the resulting rankings are presented as a heuristic aid to discussion rather than a validated quantitative metric. Future progress in oncology nanomedicine will depend not only on novel materials or targeting claims, but also on disciplined product design, biologically meaningful potency assays, scalable manufacturing, patient-selection strategies, and clear integration with precision oncology.

## Introduction

Cancer therapy has always required a balance between potency and selectivity. Conventional cytotoxic drugs can inhibit tumor growth, but systemic exposure often limits dose intensity because normal tissues share many biological vulnerabilities with malignant cells. Nanomedicine was introduced into oncology to shift this balance by modifying drug distribution, improving solubility, prolonging circulation, reducing peak-related toxicity, and creating opportunities for intratumoral or intracellular delivery [1–5]. The clinical success of pegylated liposomal doxorubicin and albumin-bound paclitaxel demonstrated that formulation architecture can convert an established anticancer molecule into a product with a different therapeutic profile [6–10].

The field has evolved substantially since the first generation of liposomal and protein-bound cancer products. The current oncology nanomedicine landscape includes liposomes, lipid nanoparticles (LNPs), albumin-bound nanoparticles, polymeric nanoparticles, polymeric micelles, nanocrystals, inorganic particles, nanoemulsions, solid lipid nanoparticles (SLNs), nanostructured lipid carriers (NLCs), and biomimetic or hybrid systems [1–5, 9, 10]. These platforms are no longer evaluated only by particle size or material novelty. For a cancer nanomedicine to translate successfully, it must solve a concrete clinical and pharmaceutical problem: improving exposure at the site of disease, reducing systemic toxicity, enabling delivery of otherwise unstable payloads, improving intracellular access, supporting combination therapy, or creating a manufacturable precision-oncology platform [1, 2, 5, 11, 12].

The rise of RNA and lipid nanoparticle technologies has added a second major phase to the field. Lipid nanoparticles and related RNA delivery systems are enabling technologies for messenger RNA (mRNA) expression, small interfering RNA (siRNA)-mediated gene silencing, self-amplifying RNA (saRNA), gene editing, and individualized neoantigen vaccines [13–24]. In oncology, this shift is especially important because cancer biology is increasingly defined by genomic, transcriptomic, immune, and microenvironmental features. Nanoparticles can therefore serve not only as carriers of cytotoxic drugs, but also as programmable delivery systems that connect tumor sequencing, antigen prediction, immune modulation, and patient-specific therapy [19–24].

At the same time, the clinical translation of cancer nanomedicine remains challenging. High levels of particle accumulation in small-animal tumors do not guarantee meaningful drug exposure in human tumors, and active targeting claims often do not translate into improved clinical outcomes [25–34]. Tumor perfusion, vascular permeability, lymphatic drainage, extracellular matrix (ECM) density, macrophage content, stromal composition, and prior therapy can vary markedly across tumor types and patients. Manufacturing scale-up, stability, sterility, potency testing, and product comparability add further development barriers [35–41]. The attrition record makes the point more forcefully than any single mechanism: transferrin-targeted siRNA nanoparticles, prostate-specific membrane antigen (PSMA)-targeted docetaxel particles, human epidermal growth factor receptor 2 (HER2)-targeted liposomal doxorubicin, cyclodextrin-camptothecin conjugates, and a liposomal microRNA mimic all reached patients on the strength of strong preclinical tumor accumulation, and none of them became a product [42–47].

This review reframes cancer nanomedicine from a development and translation perspective. Rather than treating nanoparticles as a single technology class, it examines how platform design, payload selection, tumor biology, manufacturing, analytical control, and clinical strategy must be integrated. The review is intended for oncology researchers, formulation scientists, translational drug developers, clinical investigators, and regulatory or quality professionals involved in moving nanoparticle-based oncology products from concept to clinical use.

## Review scope and approach

This article is a narrative review focused on pharmaceutical nanoparticle platforms relevant to oncology. The primary emphasis is on the period from 2010 to 2026, with earlier foundational examples included when they are essential for understanding approved products or platform evolution. The review integrates peer-reviewed literature, product-label information, regulatory guidance, clinical trial examples, and development-science concepts. It is not presented as a systematic review or meta-analysis; instead, it synthesizes development patterns, translational barriers, and practical design considerations for cancer nanomedicine.

The manuscript focuses on cancer-related applications of liposomes, lipid nanoparticles, albumin-bound nanoparticles, polymeric nanoparticles, polymeric micelles, nanocrystals, inorganic particles, nanoemulsions, solid lipid nanoparticles, nanostructured lipid carriers, extracellular vesicles (EVs), hybrid systems, and RNA/LNP platforms. Each of these classes is discussed in its own subsection of Sect. "Platform-specific development profiles" rather than being named only in the scope statement. Antimicrobial and non-oncology examples are excluded except where they provide relevant platform lessons for manufacturing, RNA delivery, or analytical control. The article also distinguishes approved or commercially relevant oncology products from early preclinical systems because approval, clinical adoption, commercial survival, and broad patient access are different outcomes, as summarized in Table 1.

**Table 1:** Oncology-relevant nanoparticle platforms and their dominant development logic

Platform	Dominant oncology function	Typical payloads	Primary translation risk
Liposomes	Pharmacokinetic modification, drug retention, controlled release, toxicity reduction	Doxorubicin, daunorubicin/cytarabine, irinotecan, other cytotoxics	Drug leakage, complement activation, release-method relevance, scale-up comparability [6–10, 36]
Lipid nanoparticles	Intracellular delivery of nucleic acids and immune-modulating payloads	mRNA, siRNA, saRNA, gene-editing components, encoded antigens	Endosomal escape, tissue targeting, lipid impurity, potency assay design, immunogenicity [13–24]
Albumin-bound nanoparticles	Solubilization and protein-associated delivery of hydrophobic anticancer drugs	Paclitaxel, sirolimus-like drugs	Free drug control, protein aggregation, infusion compatibility, batch comparability [48–53]
Polymeric nanoparticles	Controlled release, local delivery, payload protection, combination delivery	Cytotoxics, small molecules, proteins, immunomodulators, complexed RNA	Burst release, residual solvent, polymer heterogeneity, manufacturing scale-up [9, 10, 54]
Polymeric micelles	Solubilization of hydrophobic oncology drugs	Taxanes and other poorly soluble cytotoxics	Dilution instability, premature release, commercial viability [54, 55]
Nanocrystals	Dissolution-rate enhancement for very poorly soluble drugs without a carrier excipient	Crystalline drug only, stabilized by surfactant or polymer	Ostwald ripening, crystal-form change on milling, redispersion after drying, limited targeting benefit [56]
Nanoemulsions, SLNs, and NLCs	Lipid-based solubilization and depot behavior; parenteral and local administration	Lipophilic cytotoxics and immunomodulators	Physical instability on storage, drug expulsion during lipid recrystallization, sterile processing of lipid dispersions, limited oncology clinical data [57, 58]
Inorganic particles	Imaging, radiotherapy enhancement, magnetic/thermal strategies, local therapy	Iron oxide, hafnium oxide, gold, silica and other inorganic materials	Long-term retention, metal-related toxicity, clearance uncertainty, product-specific evidence [28, 29, 37]
Hybrid/biomimetic systems	Multifunctional delivery, immune evasion, membrane-inspired targeting; includes extracellular vesicles	RNA, drugs, proteins, combinations	Complex characterization, donor and source variability, sterility, viral safety, reproducibility, absence of agreed potency standards [1, 2, 35, 59]

Platform classification is kept consistent across the text, tables, and figures. Nine classes are used throughout: liposomes; lipid nanoparticles; albumin-bound nanoparticles; polymeric nanoparticles; polymeric micelles; nanocrystals; lipid-based emulsions and particles (nanoemulsions, SLNs, and NLCs); inorganic particles; and biological or hybrid carriers (extracellular vesicles, membrane-coated particles, and lipid-polymer hybrids). Dendrimers and polyplexes are treated within the polymeric class. Where an earlier version of this manuscript used different groupings in different tables, the classification has been harmonized.

Table 1 is deliberately a map rather than a summary of evidence. It shows that the platforms differ less in what they are made of than in where their development usually breaks: leakage and release for liposomes, intracellular delivery for LNPs, free drug and protein behavior for albumin systems, process control for polymeric systems, and characterization and sourcing for biological carriers. Sects. "Platform-specific development profiles" and "Tumor biology barriers as a sequential delivery cascade" take each of these in turn.

## From passive carriers to precision oncology platforms

### The EPR effect as a variable opportunity

Early cancer nanomedicine was strongly influenced by the enhanced permeability and retention effect. The concept proposed that macromolecules and nanoparticles can preferentially accumulate in tumors because tumor vasculature is abnormal and lymphatic drainage is impaired [25–27]. This biological rationale supported the development of long-circulating liposomes, polymeric micelles, albumin-bound systems, and other carrier technologies. However, later clinical and translational work showed that the EPR effect is heterogeneous and cannot be assumed to operate uniformly across tumor types, lesions, or patients [27–29].

This heterogeneity explains why a nanomedicine cannot be justified by nanoscale size alone. A particle that accumulates in one tumor model may fail in a poorly perfused or highly stromal human tumor. Similarly, attaching a ligand to a nanoparticle may increase cellular internalization under selected conditions but does not guarantee increased whole-tumor localization or clinical benefit [28, 29, 33, 34]. Contemporary cancer nanomedicine development therefore requires a more disciplined chain of evidence: the platform must address a defined clinical problem, deliver the relevant payload to the intended biological compartment, demonstrate meaningful potency, retain manufacturability at clinical scale, and show differentiation from simpler alternatives [1, 2, 9, 10].

### Active targeting: cellular binding is not tumor accumulation

The single most persistent confusion in this field is between two different measurements. Cell-level binding is measured in vitro as the fraction of a ligand-bearing particle population that attaches to and is internalized by target cells in culture. Tumor-site enrichment is measured in the whole animal as the fraction of the administered dose that ends up in tumor tissue. Adding a targeting ligand reliably improves the first and rarely improves the second. The classic demonstration used anti-HER2 immunoliposomes: ligand attachment increased internalization by tumor cells several-fold, but total tumor localization was no different from the untargeted liposome, because both particles arrived through the same vascular route [34]. Targeting changed where the particle went after it arrived, not how much of it arrived.

This distinction has practical consequences for how a development program should read its own data. A ligand that improves internalization is worth pursuing when the payload has to reach the cytosol and cannot enter cells on its own, which is why it matters for RNA and for agents that act intracellularly. It is much harder to justify when the payload is a small-molecule cytotoxic that will diffuse into cells anyway once it is released in the interstitium. Programs should therefore state which of the two effects they are claiming, and support the claim with the matching measurement: flow cytometry and confocal imaging for binding and uptake, and quantitative biodistribution or imaging for tumor accumulation [28, 33].

### What the clinical failures have shown

Reviews of this field tend to describe approved products in detail and mention failures in passing. That is the wrong balance, because the candidates that reached patients and stopped there are the closest thing the field has to a controlled experiment on what preclinical tumor accumulation actually predicts. Several instructive examples are summarized in Table 2.

**Table 2:** Representative oncology nanomedicine candidates with strong preclinical performance that did not translate, and the development lesson from each

Candidate	Platform and target	Clinical outcome	Development lesson
CALAA-01	Cyclodextrin polymer nanoparticle; transferrin-targeted siRNA against RRM2	Phase I in solid tumors; rapid clearance, exposure at the top human dose comparable to low doses in animals; development discontinued	Interspecies scaling of nanoparticle pharmacokinetics is unreliable; a targeting ligand does not compensate for a carrier that disassembles quickly in circulation [42]
BIND-014	PSMA-targeted polymeric (PLA-PEG) docetaxel nanoparticle	Tolerable in phase I with a docetaxel-distinct pharmacokinetic profile, but phase 2 activity in castration-resistant prostate cancer and in lung cancer did not exceed conventional docetaxel; program terminated	A differentiated pharmacokinetic profile is not by itself a clinical advantage; the comparator is the free drug, not the untargeted particle [43, 44]
MM-302	HER2-targeted antibody-liposomal doxorubicin conjugate	Encouraging phase I activity in anthracycline-naive patients and confirmed tumor deposition by radiolabeled imaging, but the randomized HERMIONE trial was stopped for futility	Demonstrating that the particle reaches the tumor is necessary but not sufficient; the released payload still has to outperform the available alternatives [45]
CRLX101	Cyclodextrin-polymer camptothecin nanopharmaceutical	Tolerable with signals of activity in early trials, but randomized studies did not establish benefit over standard care	Sustained release of a potent cytotoxic changes the toxicity profile more predictably than it changes efficacy [46]
NK105	Polymeric micellar paclitaxel	Phase III in metastatic breast cancer did not meet its non-inferiority endpoint against conventional paclitaxel, despite reduced neuropathy	Micelle dilution behavior after infusion governs the delivered exposure; a tolerability gain can be lost if efficacy is not preserved [60]
MRX34	Liposomal microRNA-34a mimic	Phase I terminated after severe immune-mediated adverse events	For nucleic-acid payloads, innate immune activation is a dose-limiting product attribute and has to be characterized as such, not treated as an incidental finding [47]

Read together, these programs point at the same three problems. First, the animal-to-human step for nanoparticle pharmacokinetics is weaker than for small molecules, because clearance is governed by the mononuclear phagocyte system, which differs substantially between species. Second, the relevant comparator in an oncology trial is the conventional formulation of the same drug, and improving on it requires either a real efficacy gain or a toxicity reduction large enough to change practice. Third, biological activity of the payload must be demonstrated in patients, not inferred from particle properties. These themes reappear in Sects. "Tumor biology barriers as a sequential delivery cascade", Development strategy: QTPP, CQAs, and control strategy" and "Clinical translation and evidence generation".

The transition to precision oncology has expanded the possible role of nanoparticles. Genomic testing, neoantigen prediction, immune profiling, and molecular tumor classification create new therapeutic payloads and patient-selection opportunities. RNA/LNP platforms can encode antigens, silence genes, express immunomodulatory proteins, or deliver gene-editing machinery [13–24]. These opportunities require development teams to integrate tumor biology with product quality. For example, an individualized mRNA vaccine is not only an immunology product; it is also a manufacturing, release-testing, cold-chain, potency, sterility, and turnaround-time challenge [19–24].

## Platform-specific development profiles

This section treats each platform class in the same order: what the platform is and what it does, which oncology products have been approved and when, how they are given, what the clinical evidence supports, and what limits the platform in development. Approved products are consolidated in Table 3 and platform-level advantages and disadvantages in Table 4, so the text can concentrate on interpretation rather than repeating tabulated facts.

**Table 3:** Approved and commercially relevant oncology nanomedicines, organized by platform

Product (platform)	Formulation characteristics	First approval	Route and oncology use	Clinical evidence and limitation
Doxil / Caelyx (liposome)	PEGylated liposome, ~ 80–100 nm, ammonium sulfate remote loading of doxorubicin, HSPC/cholesterol/PEG-DSPE	1995 (US)	Intravenous; ovarian cancer, Kaposi sarcoma, multiple myeloma	Reduced cardiotoxicity relative to conventional doxorubicin; limited by palmar-plantar erythrodysesthesia and infusion reactions attributable to complement activation [6–8, 61, 62]
Abraxane (albumin-bound)	Paclitaxel bound to human serum albumin, ~ 130 nm nominal particle, reconstituted suspension, no Cremophor EL	2005 (US)	Intravenous; metastatic breast cancer, non-small cell lung cancer, pancreatic cancer	Higher response rate than solvent-based paclitaxel in breast cancer and a survival gain with gemcitabine in pancreatic cancer; the particle dissociates rapidly after infusion, so the benefit is largely a solvent and exposure effect [48–50]
Onivyde (liposome)	Liposomal irinotecan sucrosofate, ~ 110 nm, remote loading, prolonged conversion to SN-38	2015 (US)	Intravenous; metastatic pancreatic adenocarcinoma with fluorouracil and leucovorin	Survival benefit after gemcitabine failure; exposure is tied to intratumoral conversion of the prodrug and to tumor deposition, which varies between lesions [51, 63]
Vyxeos (liposome)	Liposome co-encapsulating daunorubicin and cytarabine at a fixed 1:5 molar ratio	2017 (US)	Intravenous; therapy-related acute myeloid leukemia and AML with myelodysplasia-related changes	Overall survival benefit over the standard 7 + 3 regimen in the eligible population; the product’s value depends on maintaining the drug ratio in vivo, which constrains release specifications [52]
Fyarro (albumin-bound)	Sirolimus bound to human serum albumin, injectable suspension	2021 (US)	Intravenous; advanced malignant perivascular epithelioid cell tumor	Durable responses in a rare tumor with no prior standard; approval in a small population limits the commercial base and the comparative evidence [53]
Genexol-PM / Apealea (polymeric micelle)	Paclitaxel in a block-copolymer or XR-17 micelle, solvent-free	2007 (Korea); 2018 (EU)	Intravenous; breast, lung, and ovarian cancer depending on region	Allows higher paclitaxel doses without solvent-related premedication, but comparative survival benefit has not been established and the EU marketing authorization was subsequently withdrawn [54, 55]
NanoTherm (inorganic)	Aminosilane-coated superparamagnetic iron oxide dispersion for intratumoral instillation	2010 (EU, device)	Intratumoral; recurrent glioblastoma, used with an alternating magnetic field and radiotherapy	Prolonged survival in a single-arm study; local administration bypasses systemic delivery barriers but restricts use to accessible, imageable lesions [64]
Hensify / NBTXR3 (inorganic)	Crystalline hafnium oxide nanoparticles, ~ 50 nm, injected directly into the tumor as a radioenhancer	2019 (EU, device)	Intratumoral; locally advanced soft-tissue sarcoma, with preoperative radiotherapy	Doubled the pathological complete response rate versus radiotherapy alone in a randomized phase 2–3 trial; the particle is inert and non-therapeutic on its own, so benefit is entirely radiation-dependent [65]
Onpattro (lipid nanoparticle, non-oncology)	Ionizable lipid, DSPC, cholesterol, PEG-lipid, encapsulated siRNA; included as the reference LNP approval	2018 (US)	Intravenous; hereditary transthyretin amyloidosis	Establishes that systemic siRNA/LNP delivery is a viable regulated product class, but efficacy depends on hepatocyte uptake via apolipoprotein E, which is exactly the property oncology applications must overcome [66, 67]

**Table 4:** Platform-level advantages and disadvantages for oncology use

Platform class	Principal advantages	Principal disadvantages	Typical route and current oncology position
Liposomes	Established regulatory precedent and class-specific guidance; long circulation with PEGylation; remote loading gives high drug-to-lipid ratios; reduces exposure of sensitive normal tissue	Drug leakage on storage and after dosing; complement activation and infusion reactions; intact vesicle penetrates tumor tissue poorly; release methods are hard to make discriminatory	Intravenous; four approved oncology products, the most mature platform in the field
Lipid nanoparticles	The only practical way to deliver intact RNA into the cytosol at scale; programmable payload; rapid sequence-to-product turnaround	Strong hepatic tropism after intravenous dosing; endosomal escape of only a few percent; ionizable-lipid impurities; cold-chain burden; innate immune activation	Intravenous, intramuscular, intratumoral, or intranodal; no approved oncology product, but randomized vaccine data are emerging
Albumin-bound nanoparticles	Avoids solvent-related toxicity and premedication; endogenous and biodegradable carrier; strongest comparative clinical evidence of any nanoparticle oncology class	Particle dissociates within minutes, so it is not the circulating species; protein aggregation and reconstitution performance are critical; mechanism is often overstated	Intravenous after reconstitution; two approved oncology products
Polymeric nanoparticles	Highly tunable release, degradation rate, and architecture; can co-deliver combinations; suitable for local and depot delivery	Burst release; residual solvent and monomer; polymer polydispersity is intrinsic; scale-up changes particle properties; no approved oncology product	Intravenous or local; investigational, with several terminated late-stage programs
Polymeric micelles	Efficient solubilization of taxanes without solvent; simple composition; reduced neuropathy in comparative studies	Dissociate below the critical micelle concentration after infusion; premature release; efficacy non-inferiority not demonstrated; limited commercial durability	Intravenous; approved in Korea, authorization withdrawn in the European Union
Nanocrystals	No carrier excipient and therefore a simple impurity profile; large dissolution-rate gain; straightforward, well-understood manufacture	No encapsulation, protection, circulation, or targeting benefit; Ostwald ripening; crystal-form change during milling; poor redispersion after drying	Mainly oral or local; mature technology but no intravenous oncology product
Nanoemulsions, SLNs, and NLCs	Built from excipients with long parenteral use; good tolerability; suitable for depot and local release of lipophilic agents	Lipid recrystallization expels drug during storage; relatively low drug loading; difficult to sterilize; little oncology clinical data	Local, depot, or topical; investigational in oncology
Inorganic particles	Physical mechanisms not available to organic carriers (radioenhancement, magnetic hyperthermia, imaging); no pharmacological activity of their own, which simplifies potency	Not biodegradable, so long-term retention must be characterized; benefit depends entirely on the co-applied energy source; narrow eligible population	Intratumoral; two products approved in the European Union under the device pathway
Extracellular vesicles and hybrid carriers	Endogenous membrane with low immunogenicity; native surface proteins support cell-to-cell transfer; long circulation relative to comparable liposomes	Donor and passage variability; no consensus isolation, identity, or potency standard; low yield; cannot be terminally sterilized; filtration overlaps the vesicle size range	Intravenous or local; earliest-stage class, with first-in-human studies underway

Approved oncology nanomedicines provide the most reliable lessons because they connect formulation design to clinical, regulatory, and commercial outcomes. Pegylated liposomal doxorubicin demonstrated that liposomal encapsulation and PEGylation can change doxorubicin pharmacokinetics and toxicity patterns [6–8, 61]. Albumin-bound paclitaxel demonstrated that a protein-associated nanoparticle can improve delivery of a poorly soluble anticancer drug without conventional solvent systems [48–50]. Liposomal irinotecan and fixed-ratio liposomal daunorubicin/cytarabine illustrate how formulation design can support specific dosing, exposure, and combination-ratio objectives [51, 52]. Albumin-bound sirolimus particles illustrate continuing clinical relevance of albumin-associated nanoparticle products in rare tumors [53], as summarized in Table 3.

Two patterns in Table 3 are worth stating directly. The systemically administered products are all reformulations of established cytotoxics, and each of them earned approval by changing tolerability, solubility, or dosing rather than by delivering a new mechanism. The two inorganic products, by contrast, are administered directly into the lesion and are regulated as devices in the European Union. Local administration removes the accumulation and penetration problems that dominate systemic nanomedicine, which is a large part of why those two products exist. This distinction is carried through the maturity assessment in Sect. "Development-to-translation matrix for cancer nanomedicine".

The main lesson from these products is that successful cancer nanomedicines usually solve practical and clinically meaningful problems. They are not approved because they are nanoparticles; they are approved because the formulation changes how the active ingredient is delivered, tolerated, dosed, or combined. The second lesson is that approval does not automatically ensure broad commercial success. Some products or regional approvals may be discontinued, withdrawn, or limited by clinical competition, manufacturing cost, or modest differentiation [5, 55].

Therefore, review articles and development programs should not treat all approved nanoparticles as equally mature. Product maturity depends on current market presence, breadth of indication, physician adoption, manufacturing robustness, patent or generic status, supply reliability, and clinical advantage over alternatives.

Table 4 is organized so that the disadvantage column reads as a development agenda rather than a list of shortcomings. In every case the limiting property is a consequence of the same physical design that creates the advantage: PEGylation buys circulation time and brings complement activation with it, a solid lipid matrix slows release and expels drug as it recrystallizes, and an ionizable lipid enables endosomal escape and also recruits apolipoprotein E to the particle surface. Platform selection is therefore a choice of which problem to accept, and the subsections that follow discuss each choice in turn.

### Liposomes

Liposomes are phospholipid vesicles that can retain hydrophilic drugs in an aqueous core, associate lipophilic drugs with a bilayer, or retain amphipathic drugs using remote-loading gradients [6–8, 36]. In oncology, their main functions are pharmacokinetic modification, toxicity reduction, prolonged circulation, and controlled release. Liposomes can reduce exposure of sensitive normal tissues to free cytotoxic drug, but they also introduce new development questions: how much drug is free versus encapsulated, how rapidly the drug leaks during storage and after administration, how the liposome interacts with plasma proteins and the mononuclear phagocyte system, and how the product can be manufactured reproducibly [6–10, 36].

Four liposomal oncology products define the clinical experience with this platform. Pegylated liposomal doxorubicin, approved in 1995, established that encapsulation plus PEGylation can shift an anthracycline away from cardiac tissue at the cost of a new dose-limiting toxicity in the skin [6–8, 61]. Liposomal irinotecan, approved in 2015, showed that a liposome can serve as a slow-conversion depot for a prodrug and extend survival in a setting where free irinotecan had not [51]. Fixed-ratio liposomal daunorubicin and cytarabine, approved in 2017, showed that the vesicle can be used to hold two drugs at a synergistic ratio in circulation, which turns the drug-to-drug ratio into a release specification [52]. All three are given intravenously. The failed HER2-targeted liposomal doxorubicin program adds the counterpoint that decorating the same vesicle with a targeting antibody did not convert tumor deposition into clinical benefit [45].

Liposome CQAs include particle size and distribution, lamellarity, lipid composition, cholesterol content, PEGylation, drug-to-lipid ratio, encapsulation efficiency, free drug, leakage rate, osmolality, pH, sterility, endotoxin, and stability [35–41, 68, 69]. Oncology development should also consider whether in vitro release methods are discriminatory and biologically meaningful. A release method that is convenient but not clinically relevant may fail to support product understanding or comparability.

Two liposome-specific risks deserve more attention than they usually receive. The first is complement activation. Pegylated liposomes can trigger complement activation-related pseudoallergy, which presents as an infusion reaction and is a property of the particle surface rather than of the drug [62]. It is worth screening for during formulation selection, not after the first clinical infusion reaction. The second is that a liposome is a thermodynamically metastable structure, so drug leakage during storage and after administration is a continuum rather than a pass-or-fail attribute; the specification has to be set against a release method that actually discriminates between batches that behave differently in circulation [36].

### Albumin-bound nanoparticles

Albumin-bound nanoparticles are attractive because albumin is endogenous, biodegradable, and capable of binding hydrophobic molecules. In oncology, albumin-bound paclitaxel and albumin-bound sirolimus particles show that protein-associated delivery can be clinically meaningful when it improves solubility, infusion compatibility, or exposure [48–53]. However, albumin-based products require careful control of protein aggregation, free drug, particle-size distribution, reconstitution performance, infusion compatibility, and batch comparability.

The approved products are albumin-bound paclitaxel, first approved in 2005 for metastatic breast cancer and later extended to non-small cell lung cancer and pancreatic cancer, and albumin-bound sirolimus, approved in 2021 for malignant perivascular epithelioid cell tumor [48–53]. Both are given intravenously after reconstitution. The clinical evidence for albumin-bound paclitaxel is the strongest of any nanoparticle oncology product: a higher response rate than solvent-based paclitaxel in breast cancer and a survival gain in combination with gemcitabine in pancreatic cancer [48–50]. The main development constraint is that the particle is not the delivered entity. It dissociates within minutes of infusion into drug-bound albumin complexes, so specifications on particle size describe the reconstituted product rather than the circulating species, and the analytical package has to cover both.

A common misconception is that albumin-bound delivery automatically produces active tumor targeting through albumin receptors or tumor albumin metabolism. Although albumin biology can contribute to distribution, clinical success still depends on product-specific pharmacokinetics, tumor type, dosing regimen, toxicity profile, and comparative efficacy. Development claims should therefore be framed around evidence rather than mechanistic overinterpretation.

### Polymeric nanoparticles and polymeric micelles

Polymeric nanoparticles provide high design flexibility because polymer chemistry, molecular weight, degradation rate, surface modification, and particle architecture can be adjusted. They can be designed for controlled release, local delivery, combination therapy, or payload protection [9, 10, 54]. However, this tunability also creates translation risk. Residual solvent, polymer heterogeneity, burst release, degradation products, sterile processing, and reproducibility often become major barriers when moving from small-scale preclinical batches to clinical manufacturing.

Polymeric micelles are particularly useful for hydrophobic anticancer agents because amphiphilic block copolymers can self-assemble into structures with a hydrophobic core and hydrophilic corona [54]. Their major vulnerability is dilution instability after intravenous administration. If micelles dissociate below their critical micelle concentration, free drug can be released prematurely, reducing the intended delivery advantage. Micellar oncology products should therefore be evaluated through dilution-stability, release, and clinically relevant exposure data, not only particle-size measurements.

No polymeric nanoparticle has been approved for oncology in the United States or the European Union. Polymeric micellar paclitaxel has been approved in Korea since 2007 and was authorized in the European Union in 2018 before the marketing authorization was withdrawn [54, 55]. The phase III comparison of a micellar paclitaxel against conventional paclitaxel is the most informative result available for this class: neuropathy was reduced, but non-inferiority for efficacy was not demonstrated [60]. Together with the terminated PSMA-targeted docetaxel program, this points to a consistent conclusion. The class works well as a solubilization and tolerability technology and has not yet shown that controlled release from a polymer improves oncology outcomes [43, 44, 46].

### Nanocrystals

Nanocrystals are the simplest platform in this review because there is no carrier. The drug itself is milled or precipitated to submicron size and stabilized with a surfactant or polymer at the particle surface. Reducing particle size increases surface area and therefore dissolution rate, which is what makes very poorly soluble compounds formulable at all [56]. For oncology this matters mainly on the input side of development: a nanocrystal suspension is often the fastest way to get a poorly soluble candidate into preclinical and early clinical study without committing to a lipid or polymer platform.

The class has produced marketed products in other therapeutic areas but no approved intravenous oncology product, and the reasons are informative. A nanocrystal has no encapsulation, so it provides no protection from metabolism, no circulation-time benefit, and no targeting rationale beyond dissolution. Its development risks are physical: Ostwald ripening during storage, polymorphic or amorphous conversion caused by the energy input during milling, and aggregation on redispersion after drying. Particle size, crystalline form by X-ray powder diffraction, and redispersibility are therefore the attributes that carry the control strategy [56]. For oral or local oncology applications nanocrystals remain a reasonable choice; for a systemic product where distribution is the objective, they answer a different question than the rest of the platforms discussed here.

### Nanoemulsions, solid lipid nanoparticles, and nanostructured lipid carriers

These three are usefully treated together because they share a lipid matrix and differ in its physical state. A nanoemulsion holds the drug in a liquid oil droplet stabilized by surfactant. A solid lipid nanoparticle replaces the oil with a lipid that is solid at body temperature, which slows diffusion out of the particle. A nanostructured lipid carrier blends solid and liquid lipids deliberately to create an imperfect crystal lattice with more room for drug [57, 58]. All three are made from excipients with long parenteral use, which is a genuine regulatory advantage over newer synthetic materials.

None has yet produced an approved systemic oncology product, and the limiting factor is physical rather than biological. The solid lipid matrix tends to recrystallize into a more ordered form during storage, and the drug is expelled as it does so, which shows up as a gradual loss of encapsulated drug over shelf life. Lipid dispersions are also awkward to sterilize: terminal heat can disrupt the particle and filtration is difficult for the larger size distributions typical of the class. Drug loading is generally lower than for liposomes at the same lipid content [57]. These systems are most persuasive in oncology for local, depot, or topical use, and for lipophilic immunomodulators where a slow local release profile is the point.

### Inorganic nanoparticles

Inorganic particles are the one class where two oncology products have reached patients, and both did so by abandoning systemic delivery. Aminosilane-coated iron oxide particles are instilled directly into recurrent glioblastoma and heated by an alternating magnetic field, with radiotherapy; the approach prolonged survival in a single-arm study and was approved in the European Union as a device in 2010 [64]. Crystalline hafnium oxide particles are injected into soft-tissue sarcomas before preoperative radiotherapy, where their high atomic number amplifies local energy deposition; the randomized Act.In.Sarc trial roughly doubled the pathological complete response rate compared with radiotherapy alone, and the product was approved as a device in 2019 [65].

Both products make the same design trade. By giving up systemic administration they eliminate the accumulation and penetration problems that dominate the rest of this review, and they accept a narrow eligible population in exchange. Neither particle is pharmacologically active on its own, which simplifies the potency question but makes the product entirely dependent on the co-administered radiation. The unresolved issue for the class is what happens to material that is not cleared. Gold, silica, and hafnium oxide are not biodegradable, and long-term retention in liver, spleen, and lymph nodes has to be characterized over a horizon much longer than a typical oncology trial [28, 29, 37]. Iron oxide has an advantage here because iron enters physiological handling pathways, which is also why ferumoxytol has been usable as an imaging agent to study macrophage content and nanoparticle deposition in human tumors [63].

### Extracellular vesicles and other biological or hybrid carriers

Extracellular vesicles are membrane-bound particles released by cells, typically 30–200 nm, carrying lipids, proteins, and nucleic acids from the parent cell. Their appeal in oncology is that they are endogenous. They are not recognized as foreign in the way synthetic particles are, they carry native membrane proteins that can support cell-to-cell transfer, and mesenchymal stromal cell-derived vesicles in particular circulate longer than comparable liposomes [70]. The most developed oncology example is a mesenchymal stromal cell-derived exosome loaded with siRNA against mutant KRAS, which suppressed tumor growth and improved survival in pancreatic cancer models and progressed into a phase I trial [71].

The translational hurdles are concentrated in manufacturing and characterization rather than in biology, and they are more severe than for any synthetic platform. Production starts with a living cell culture, so the product inherits donor variability, passage-dependent drift, and media-derived vesicle contamination; two batches from the same cell line at different passages are not necessarily the same product. Isolation methods do not agree with each other: ultracentrifugation, size-exclusion chromatography, and precipitation yield preparations that differ in purity, vesicle subpopulation, and co-isolated protein, and there is no consensus reference method. Yields are low, so scaling to clinical quantities means scaling cell culture rather than scaling a mixing step. There is no agreed potency assay, and identity is currently established through a combination of particle count, membrane-marker profile, and cargo measurement rather than a single defining attribute. The MISEV2023 framework provides reporting standards for characterization but is not a specification, and the gap between publishable characterization and a release testing package remains wide [59, 70]. Sterility adds a further constraint, since filtration overlaps the vesicle size range and terminal sterilization is not an option.

Membrane-coated synthetic particles and lipid-polymer hybrids sit in the same category for development purposes. They combine a synthetic core, which is controllable, with a biological surface, which is not, and the analytical package has to cover both. Until a routine potency assay and a reproducible sourcing model exist, this class should be described as scientifically promising and commercially immature, which is how it is scored in Sect. "Development-to-translation matrix for cancer nanomedicine" [1, 2, 35].

### Lipid nanoparticles and RNA oncology platforms

Lipid nanoparticles are now central to nucleic-acid delivery. Most clinically relevant RNA/LNP formulations include an ionizable lipid, helper phospholipid, cholesterol, PEG-lipid, and RNA payload [13–18]. The ionizable lipid supports RNA complexation during formulation and contributes to endosomal escape after cellular uptake. Helper lipids and cholesterol influence structure and membrane behavior, while PEG-lipids control particle size and colloidal stability [13–18]. The distinction between liposomes and lipid nanoparticles in oncology applications is shown in Fig. 1.

**Fig. 1:**
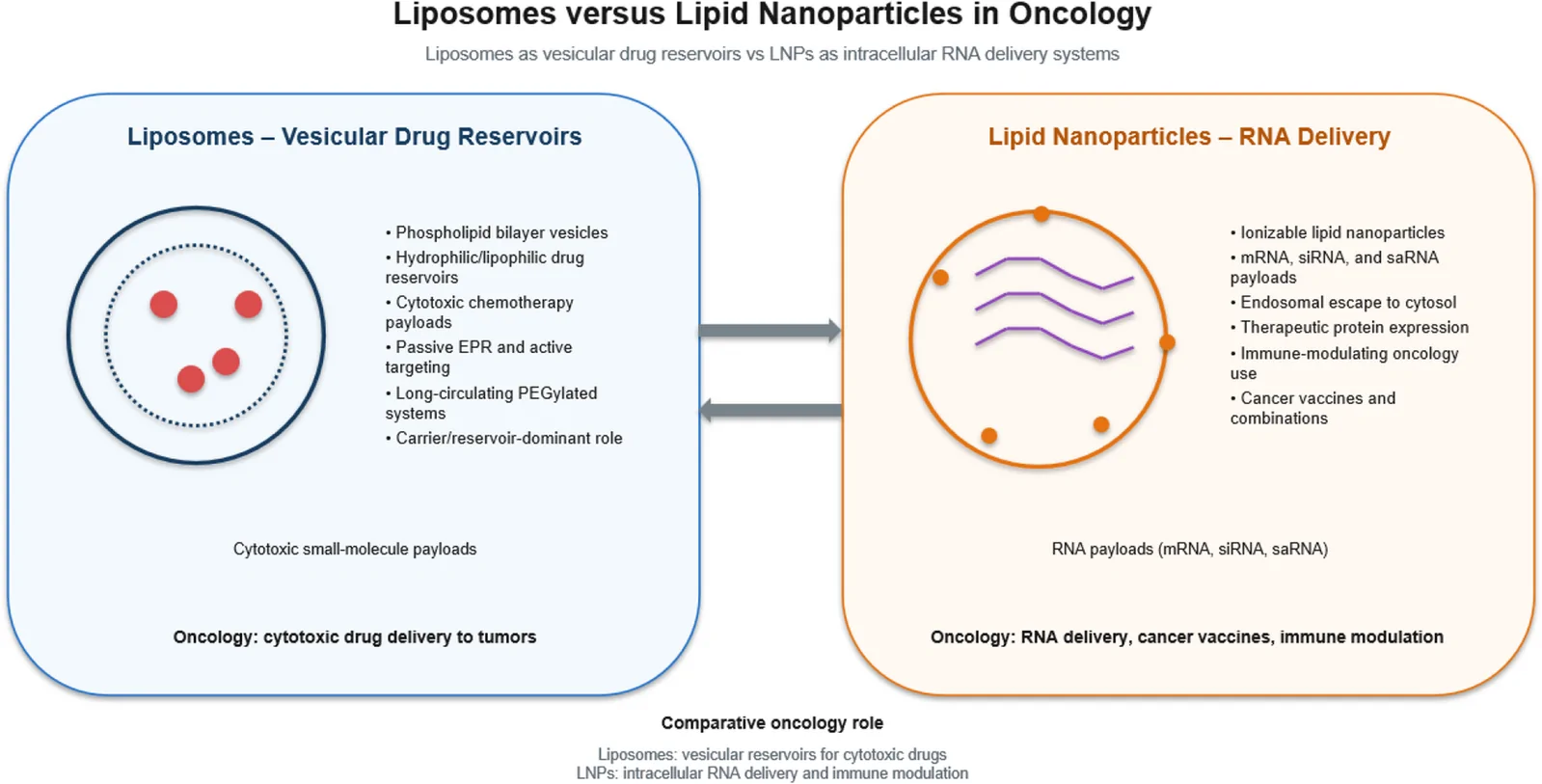
Liposomes versus lipid nanoparticles in oncology. Left: a liposome is a bilayer vesicle with an aqueous core, used as a reservoir that modifies the pharmacokinetics of an encapsulated small-molecule drug and releases it extracellularly. Right: a lipid nanoparticle (LNP) has an inverted-micellar internal structure in which nucleic acid is complexed with ionizable lipid, and its function is to deliver that payload into the cytosol of a target cell. The two architectures therefore have different critical quality attributes: drug retention and release for the liposome, and RNA integrity, encapsulation, and functional expression for the LNP. Abbreviations: LNP, lipid nanoparticle; PEG, polyethylene glycol; RNA, ribonucleic acid. Figure prepared by the authors using Microsoft PowerPoint (Microsoft 365)

#### mRNA cancer vaccines

In oncology, RNA/LNP platforms are especially relevant to individualized neoantigen vaccines, tumor-antigen expression, immune modulation, siRNA-mediated gene silencing, and future gene-editing or local-expression strategies [19–24, 72]. The KEYNOTE-942 study of individualized neoantigen therapy mRNA-4157/V940 plus pembrolizumab in resected melanoma reported improved recurrence-free survival compared with pembrolizumab alone, supporting the broader concept that RNA-based individualized immunotherapy can be integrated with checkpoint inhibition [19]. Personalized RNA neoantigen vaccine studies in pancreatic cancer further demonstrate the feasibility of linking tumor sequencing, antigen selection, individualized manufacturing, and immune readout [20, 21]. Long-term follow-up of the pancreatic cancer cohort has since shown that vaccine-induced CD8 + T cell clones can persist for years and that patients who mounted a response had longer recurrence-free survival, which is the first evidence that the immunological readout used for these products tracks a durable clinical effect [21].

Two distinct product concepts sit inside the term mRNA cancer vaccine, and they have different development profiles. An individualized neoantigen vaccine encodes mutations found in one patient’s tumor, so every batch is a batch of one and the manufacturing, release, and chain-of-identity workflow is the product. A shared-antigen vaccine encodes tumor-associated antigens common to a patient population, which makes it an off-the-shelf product with conventional batch economics but requires the antigen itself to be immunogenic enough in enough patients; the FixVac approach in checkpoint-inhibitor-treated melanoma is the clearest clinical example [22–24]. A third concept, self-amplifying RNA, reduces the dose of RNA needed by encoding a replicase alongside the antigen, at the cost of a longer construct and a more complex innate immune profile [15, 17].

#### siRNA and microRNA approaches

Gene silencing follows a different logic from vaccination. Here the nanoparticle has to reach the malignant cell itself, deliver enough siRNA into the cytosol to load the RNA-induced silencing complex, and sustain knockdown long enough to matter. The technology is clinically validated in the liver, where an ionizable-lipid LNP adsorbs apolipoprotein E and is taken up by hepatocytes through the low-density lipoprotein receptor [66, 67]. Oncology targets are usually not in the liver, which is why the same chemistry has not translated. The transferrin-targeted cyclodextrin polymer siRNA particle demonstrated RNA interference in human tumor biopsies but was cleared too quickly to reach a therapeutic exposure [42].

MicroRNA mimics and inhibitors are a related but distinct payload class. A single microRNA regulates many transcripts, which is attractive when the objective is to reset a phenotype rather than silence one gene, and correspondingly risky because off-target regulation is intrinsic to the mechanism rather than an impurity. A liposomal microRNA-34a mimic reached phase I in solid tumors and was terminated after severe immune-mediated adverse events [47]. For both siRNA and microRNA products, the practical consequence is that innate immune activation, sequence-dependent off-target effects, and duration of knockdown belong in the product characterization package from the beginning [16].

#### Non-lipid and hybrid non-viral carriers for RNA

LNPs dominate this field, but they are not the only non-viral option, and the alternatives address the LNP’s specific weaknesses. Polymeric carriers complex RNA electrostatically into polyplexes, most commonly using polyethylenimine or poly(beta-amino ester) chemistries. Their advantages are chemical tunability, scalable synthesis, and freedom from the cold-chain constraints of ionizable-lipid formulations; their historical weakness is cytotoxicity at the cationic charge densities needed for efficient complexation. Recent work has addressed this by modifying low-molecular-weight polyethylenimine with cyclic disulfide monomers, which strengthens nucleic acid binding while keeping the polymer small enough to be tolerated, and has produced organ-selective transfection that depends on the route of administration rather than on a targeting ligand [73]. Applied to cancer vaccination, the same chemistry gives a transferrin receptor-associating polyplex that is picked up by recruited monocytes after subcutaneous injection and carried to draining lymph nodes, where translation and antigen presentation occur; this both increases lymph node delivery and reduces off-target liver uptake [74].

Lipopolyplexes combine the two approaches. RNA is first condensed with a polymer core and the complex is then enveloped in a lipid shell, which separates the job of protecting and condensing the payload from the job of interacting with the cell membrane. A lipopolyplex-formulated mRNA vaccine encoding tandem neoantigens has elicited strong neoantigen-specific CD8 + T cell responses and antitumor activity across several syngeneic tumor models, which supports the argument that the delivery format, not only the antigen prediction, determines how much immunity a given construct generates [75].

From a development standpoint these carriers are less mature than LNPs and carry their own control problems: polymer polydispersity is an inherent attribute rather than an impurity, residual monomer and catalyst have to be controlled, and the polymer-to-RNA ratio behaves as a critical process parameter in the same way the N/P ratio does for lipid systems. They are nonetheless the most credible near-term route to extrahepatic and lymph node-directed RNA delivery, and a review of this field that treats LNPs as the only option overstates how settled the delivery question is.

#### Extrahepatic delivery: why systemic mRNA still goes to the liver

The most important unsolved problem for systemic RNA oncology is not tumor targeting in the abstract; it is that intravenously administered LNPs go to the liver almost regardless of what is done to them. The mechanism is now reasonably well understood. Conventional ionizable-lipid LNPs acquire an apolipoprotein E-rich protein corona in plasma, and that corona is recognized by the low-density lipoprotein receptor, which is expressed at high density on hepatocytes. The particle is therefore delivered by an endogenous targeting system that the formulator did not design and cannot easily switch off [67, 76]. Hepatic first-pass uptake, splenic and macrophage capture, and rapid clearance together leave only a small fraction of the dose available to a solid tumor, and that fraction still has to complete the penetration, uptake, and endosomal escape steps described in Sect. "Tumor biology barriers as a sequential delivery cascade".

Three approaches are being pursued. The first is to change the corona rather than add a ligand: selective organ targeting adds a fifth charged lipid to a conventional four-component LNP, which shifts the adsorbed protein profile and redirects delivery to lung or spleen in a manner that is predictable from the added lipid’s charge [76, 77]. Because the spleen is an antigen-presenting organ, spleen-directed LNPs are directly useful for cancer vaccination even though they do not target tumor tissue. The second is to change the route. Intratumoral, intranodal, subcutaneous, and intradermal administration bypass hepatic first-pass entirely, and for vaccination the draining lymph node rather than the tumor is the intended site of action [73, 74]. The third is to accept the liver and choose payloads for which hepatic expression is therapeutically useful, such as secreted immunomodulators intended to act systemically.

What remains genuinely unsolved is meaningful intratumoral payload delivery after intravenous administration of an mRNA/LNP product. Achieving it would require simultaneously reducing hepatic uptake, surviving long enough in circulation to reach a poorly perfused tumor, extravasating through abnormal vasculature, penetrating the extracellular matrix, and escaping the endosome in malignant cells rather than in tumor-associated macrophages. No current platform does all five. Development programs should therefore be explicit about whether their product is a vaccine that needs to reach lymphoid tissue, a systemic protein-expression product that can use the liver, or a tumor-directed product that must solve the full cascade, because the three have different feasibility profiles and should not be discussed as one modality.

Despite this promise, RNA/LNP oncology products remain developmentally complex. The drug product must protect RNA from degradation, maintain particle integrity during storage and administration, enable cellular uptake, promote endosomal escape, produce the intended protein or gene-regulatory effect, and balance innate immune stimulation with tolerability [13–18]. Potency testing is particularly challenging because particle size or encapsulation percentage does not necessarily predict functional intracellular delivery. Key opportunities and development needs for these platforms are summarized in Table 5.

**Table 5:** RNA/LNP and programmable nanoparticle opportunities in oncology

Approach	Oncology rationale	Development needs	Translation status
mRNA neoantigen vaccines	Encode patient-specific tumor antigens to stimulate antitumor T-cell responses	Fast sequence-to-product workflow, RNA integrity, potency, sterility, cold-chain, release criteria	Clinical evidence emerging; not a conventional off-the-shelf product [19–24]
Shared-antigen mRNA vaccines	Encode common tumor antigens or immunogenic proteins	Antigen selection, immune activation balance, delivery to antigen-presenting cells	Investigational and tumor-context dependent [22–24]
siRNA/LNP or RNAi systems	Silence oncogenic, resistance, or immune-evasion pathways	Extrahepatic/tumor targeting, endosomal escape, duration of knockdown, off-target assessment	Validated in non-oncology liver delivery; oncology translation remains more difficult [16, 66]
microRNA mimics and inhibitors	Reset a regulatory network rather than silence a single transcript	Control of innate immune activation, sequence-dependent off-target regulation, dose-limiting toxicity	Early clinical experience has been limited by immune-mediated toxicity [47]
mRNA-encoded cytokines or immune modulators	Local or systemic expression of immunostimulatory proteins	Dose control, tissue specificity, cytokine toxicity, repeat dosing	Preclinical and early clinical development depending on construct
Gene-editing components	Correct or disrupt cancer-relevant genomic targets	Co-delivery, specificity, off-target editing, long-term safety	Early translational area; high regulatory and safety complexity
Polyplex and lipopolyplex RNA carriers	Extrahepatic and lymph node-directed delivery without a targeting ligand; no ionizable lipid required	Polymer polydispersity, residual monomer and catalyst, polymer-to-RNA ratio control, cytotoxicity at high charge density	Preclinical, with strong neoantigen-specific immune responses and organ-selective transfection reported [73–75]
Local/regional RNA delivery	Increase exposure in tumors or tumor-draining tissues while reducing systemic exposure	Route-specific device compatibility, retention, local inflammation, release kinetics	Promising for selected accessible tumors

The common thread in Table 5 is that the development need shifts as the site of action moves. Products acting on lymphoid tissue need immune potency assays and rapid manufacturing; products acting on the tumor cell need evidence of cytosolic delivery; products acting systemically through an expressed protein need dose control and repeat-dose tolerability. Treating all of them as one RNA platform obscures the fact that only the first group currently has supportive randomized clinical data.

## Tumor biology barriers as a sequential delivery cascade

The most important barrier in cancer nanomedicine is not always formulation design; it is often tumor biology. Solid tumors are spatially heterogeneous, with abnormal vessels, high interstitial pressure, dense extracellular matrix, irregular lymphatic drainage, hypoxia, necrosis, macrophage infiltration, and variable stromal content [25–29]. These features influence how nanoparticles leave the bloodstream, penetrate tissue, interact with cells, and release payload. Hematologic malignancies create different challenges, including bone marrow access, circulating tumor cells, immune-cell interactions, and disease-specific microenvironments.

It is more useful to treat these barriers as a sequence than as a list. A nanoparticle has to complete five steps before it produces a biological effect: reach the tumor, move through the tissue, enter the right cells, reach the correct intracellular compartment, and release an active payload. The efficiencies multiply, so a platform that is excellent at four steps and poor at one performs like a platform that is poor overall. Framing the problem this way also makes the platform comparison tractable, because each class fails at a characteristic step rather than failing uniformly. This structure is shown in Fig. 2 and compared across platforms in Table 6.

**Table 6:** Comparative behavior of the major platform classes at each step of the delivery cascade

Barrier	Liposomes	Albumin-bound particles	Lipid nanoparticles	Polymeric particles and micelles
Protein-corona formation	Dense PEG layer limits but does not prevent opsonization; complement proteins bind the PEG surface and can trigger infusion reactions [62]	The particle is albumin, so there is no distinct corona; behavior is governed by physiological albumin handling and by drug-albumin binding	Apolipoprotein E adsorption is the dominant event and actively directs the particle to hepatocytes via the low-density lipoprotein receptor [67, 76]	Corona composition depends strongly on surface charge and hydrophobicity; cationic polyplexes bind serum proteins rapidly and can aggregate [78]
Mononuclear phagocyte clearance	Slow for pegylated vesicles, giving long circulation; accelerated blood clearance can occur on repeat dosing	Not applicable in the usual sense because the particle dissociates before it is recognized as particulate	Substantial splenic and hepatic macrophage capture in addition to hepatocyte uptake	Highly variable; micelles below the critical micelle concentration disassemble rather than being cleared as particles [54]
Tumor matrix penetration	Poor for the intact vesicle, which remains largely perivascular; the released drug penetrates further [79]	Effectively that of albumin and free drug rather than of a nanoparticle, which is an advantage in stroma-rich tumors	Poor and largely untested, since hepatic uptake limits the dose reaching the tumor in the first place	Poor for intact particles; micelles that dissociate behave as free drug [80]
Rate-limiting step overall	Steps 1–2 and the coupling between release and penetration	Steps 1–3, but the relevant species is released drug, not a particle	Steps 1 and 4; endosomal escape is the largest single inefficiency [81]	Steps 1–2, driven by dilution instability and premature release
Most informative measurement	Discriminatory release in biorelevant media plus lesion-level imaging of deposition [63]	Free versus bound drug in plasma; particle size describes only the reconstituted product	Functional potency in a relevant cell type, not encapsulation efficiency	Dilution-stability and premature-release testing across the clinically relevant concentration range

### Step 1: tumor accumulation

The EPR effect remains an important concept, but it should be treated as a variable biological opportunity rather than a universal mechanism. Across human tumors, nanoparticle accumulation may be limited by poor perfusion, vessel normalization after therapy, high stromal density, or rapid clearance by liver and spleen [27–29]. Even when particles reach the tumor, they may remain near blood vessels or be taken up by macrophages rather than malignant cells. For RNA/LNP platforms, intracellular delivery adds further barriers: endocytosis is not equivalent to cytosolic release, and endosomal escape is often inefficient [13–18].

The platforms behave differently here for reasons that are mostly about circulation time and surface chemistry. Long-circulating pegylated liposomes are the best performers, because a dense PEG layer slows opsonization and the mononuclear phagocyte system (MPS) removes them relatively slowly; this is the property that made the first generation of products viable. Albumin-bound particles behave quite differently, because they dissociate within minutes of infusion, so what accumulates is drug-bound albumin rather than a particle, and the distribution question becomes a protein transport question. Polymeric micelles occupy an intermediate position that depends on whether they remain above their critical micelle concentration after dilution into blood. Intravenously administered LNPs are the weakest, for the apolipoprotein E and low-density lipoprotein receptor reasons set out in Sect. "Extrahepatic delivery: why systemic mRNA still goes to the liver" [67, 76]. Across all of them, the fraction of an administered dose that reaches a solid tumor is low and highly variable between lesions in the same patient, which is why lesion-level imaging measurements have been more informative than average biodistribution [28, 63].

### Step 2: tissue penetration

Arriving at the tumor is not the same as being distributed within it. Solid tumors have elevated interstitial fluid pressure, which removes the pressure gradient that would otherwise drive convective transport outward from vessels, leaving diffusion as the main mechanism. Diffusion through a dense collagen and hyaluronan matrix is strongly size-dependent, so larger particles are effectively trapped in the perivascular region while the drug they carry may diffuse much further once released [79, 80]. The practical implication is uncomfortable for carrier design: for a small-molecule cytotoxic, deep tissue penetration is usually achieved by the released drug rather than by the intact carrier, which means the release step and the penetration step are coupled. Stroma-rich tumors such as pancreatic adenocarcinoma are the hardest case, and this is a plausible part of why albumin-bound paclitaxel, which delivers a rapidly released drug, performed well there while carriers designed for prolonged retention have not [49].

### Step 3: cellular uptake

Within the tumor, nanoparticles are taken up preferentially by tumor-associated macrophages and other phagocytic cells rather than by malignant cells. This is a consequence of the same phagocytic machinery that clears particles from the circulation, operating locally [29]. Whether it is a problem depends entirely on the payload. For a cytotoxic that will be released and diffuse to neighboring cells, macrophage uptake can act as a local depot. For an RNA payload that must be translated inside a specific cell type, uptake by the wrong cell is a complete loss of that fraction of the dose. This is the step at which ligand targeting can genuinely help, and it is also the step at which improved in vitro binding gets over-interpreted as improved delivery, as discussed in Sect. "Active targeting: cellular binding is not tumor accumulation" [34].

### Step 4: intracellular trafficking and endosomal escape

Endocytosis delivers the particle into an endosome, not into the cytosol. For nucleic acid payloads the particle must destabilize the endosomal membrane and release its cargo before the endosome matures into a lysosome or is recycled back to the cell surface. Quantitative imaging of siRNA-LNP trafficking showed that only a small percentage of internalized siRNA reaches the cytosol, with most of the dose returned to the extracellular space through recycling within the first hour [81]. This is the single largest inefficiency in the entire cascade and the reason ionizable lipid chemistry, rather than particle size, dominates LNP optimization. It is also the reason a potency assay for an RNA product must measure a functional consequence and not encapsulation, since encapsulation efficiency says nothing about escape efficiency [13–18].

### Step 5: functional payload release

The final step differs by payload. A liposomal cytotoxic must release free drug in a form and at a rate that produce a cytotoxic concentration in tumor tissue; release that is too slow wastes the delivered dose and release that is too fast removes the rationale for the carrier. A prodrug-carrying liposome adds a conversion step that depends on tumor enzymology and therefore varies b

etween patients [51]. An mRNA product must produce translated protein in sufficient quantity and for sufficient duration, and for a vaccine that protein must then be processed and presented. An siRNA product must load the RNA-induced silencing complex and maintain knockdown. Because these endpoints are so different, a single universal release test is not meaningful across the platform classes, and each product needs a release or potency method chosen against its own mechanism, as discussed in Sects. "Functional potency assays for RNA/LNP oncology products".

**Fig. 2:**
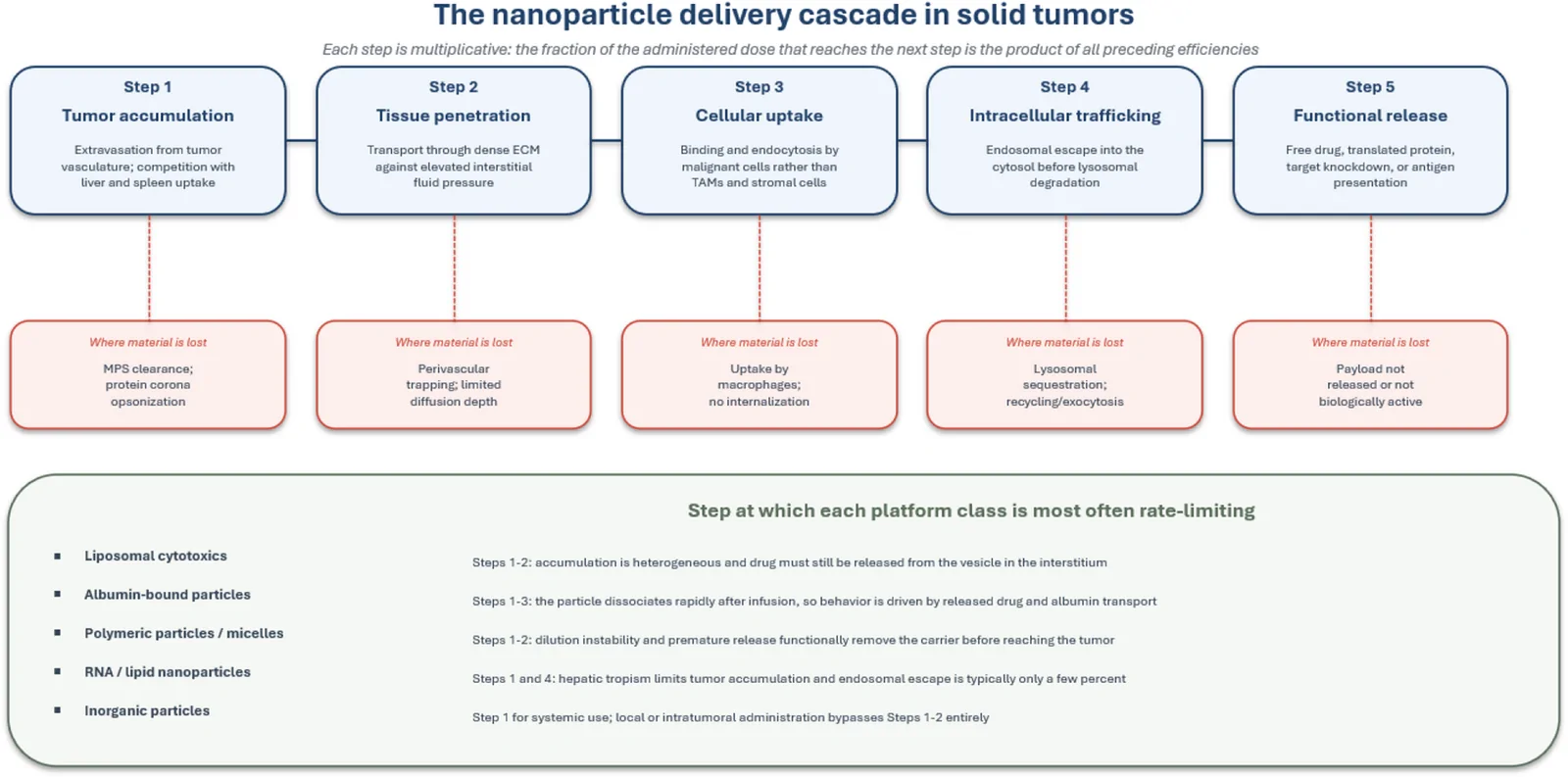
The nanoparticle delivery cascade in solid tumors. Nanoparticles must complete five sequential steps before producing a biological effect, and the efficiencies of these steps multiply. Blue boxes show each step; red boxes show the dominant mechanism of loss at that step; the lower panel indicates the step at which each platform class is most often rate-limiting. Local or intratumoral administration bypasses Steps 1 and 2. Abbreviations: ECM, extracellular matrix; MPS, mononuclear phagocyte system; TAM, tumor-associated macrophage. Figure prepared by the authors using Microsoft PowerPoint (Microsoft 365)

Protein-corona formation also changes particle identity in vivo. Once administered, nanoparticles interact with plasma proteins, complement components, lipoproteins, immunoglobulins, and extracellular matrix proteins [30–32]. The resulting biological identity can alter biodistribution, cellular uptake, immune recognition, and clearance. Surface chemistry, PEG density, charge, hydrophobicity, and particle stiffness influence these interactions. Therefore, formulation design must consider the biological environment in which the nanoparticle will operate, not only the intended composition at release.

## Development strategy: QTPP, CQAs, and control strategy

A cancer nanomedicine development program should start with a quality target product profile (QTPP). The QTPP defines the intended indication, route, dosing schedule, patient population, combination regimen, target tissue or cell type, storage condition, administration device, and expected clinical advantage. A liposomal cytotoxic product, an individualized mRNA vaccine, an albumin-bound rare-tumor drug, and an intratumoral immune-modulating nanoparticle have different QTPPs and therefore different critical quality attributes (CQAs), where a CQA is a physical, chemical, biological, or microbiological property or characteristic that must be within an appropriate limit, range, or distribution to assure the desired product quality [35–41].

### How CQAs are identified and prioritized

CQAs are not a fixed checklist that applies to every nanoparticle. They are identified by working backwards from the QTPP through a formal risk assessment, as described in ICH Q8(R2) and ICH Q9(R1) [39, 40]. In practice this means asking three questions of every measurable attribute. First, is there a plausible mechanistic link between this attribute and a clinical outcome of safety or efficacy? Second, how severe is the consequence if the attribute moves outside its range? Third, how much uncertainty is there about the relationship, given the current stage of development? Attributes that score high on severity are always critical; attributes that score high on uncertainty are provisionally treated as critical and may be downgraded as process and clinical understanding accumulate. The output is a ranked list specific to that product, not a universal set.

The ranking differs sharply between platform classes because the mechanistic link differs. For a liposomal cytotoxic, the attribute closest to clinical outcome is the free-to-encapsulated drug ratio and its change over shelf life, because free drug drives the toxicity the formulation exists to avoid; particle size matters mainly because it influences clearance and filterability. For an RNA/LNP product the hierarchy inverts. RNA integrity and functional potency sit at the top, because a particle of perfect size containing fragmented or untranslatable RNA has no activity at all, while ionizable-lipid impurities matter because reactive species can adduct to the RNA and reduce expression during storage. For an albumin-bound product, protein aggregation, free drug, and reconstitution performance dominate, because the product is administered as a suspension prepared at the bedside. For polymeric systems, residual solvent, polymer molecular weight distribution, and burst release dominate, because they are the attributes most sensitive to the manufacturing process. For extracellular vesicles, identity and purity themselves remain open questions, so characterization rather than specification is the current state of practice [59]. Table 7 sets out these differences explicitly rather than presenting one universal CQA set.

**Table 7:** Practical CQA map for oncology nanoparticle products, with platform-specific prioritization

CQA domain	Examples	Why it matters in oncology	Typical tools	Platform for which it ranks highest
Particle size/distribution	Mean size, PDI, D10/D50/D90, aggregates	Affects clearance, tumor access, filtration, and reproducibility	DLS, NTA, laser diffraction, AF4, microscopy [68, 69]	All classes, but decisive for sterile-filterable liposomes and for nanocrystals
Morphology/architecture	Vesicle, micelle, crystal, lipid/RNA particle, hybrid morphology	Confirms platform identity and supports comparability	Cryo-TEM, TEM, SEM, AFM [68, 69]	Lipid nanoparticles, where internal structure relates to encapsulation and escape
Payload association	Encapsulated/free drug, RNA encapsulation, drug-to-lipid ratio	Free drug may drive toxicity; encapsulation may drive exposure and potency	Separation methods, HPLC/UPLC, electrophoresis [35–41, 68, 69]	Liposomal cytotoxics and albumin-bound products, where free drug is the toxicity driver
Release/leakage	In vitro release, serum leakage, dilution stability	Links product quality to tumor exposure and systemic toxicity	Dialysis, sample-and-separate, flow-through, biorelevant media [36]	Liposomes and polymeric micelles; dilution stability is the defining micelle attribute
Functional potency	Cytotoxicity, expression, knockdown, immune activation, antigen presentation	Physical quality may not predict biological performance	Cell-based assays, reporter assays, cytokine/immune assays [13–24]	RNA/LNP products, where it is the single highest-ranked attribute
Chemical purity	Lipid impurities, polymer degradation, residual solvent, oxidation	Impacts safety, stability, and repeat-dose feasibility	LC-MS, HPLC, GC, GPC, impurity methods [35–41]	Lipid nanoparticles (ionizable-lipid degradants) and polymeric particles (residual solvent and monomer)
Microbiological quality	Sterility, endotoxin, bioburden strategy	Parenteral and intratumoral oncology products require robust sterile strategy	Compendial tests and validated process controls [35, 36]	All parenteral products; most constraining for extracellular vesicles and lipid dispersions that cannot be terminally sterilized
Stability	Aggregation, leakage, RNA degradation, potency loss	Storage and handling failures can change dose and clinical performance	Stability-indicating analytical and potency methods [17, 35–41, 68, 69]	RNA/LNP products, where RNA integrity and potency loss define the cold-chain requirement

Critical quality attributes should be selected based on platform mechanism and clinical use. For liposomes, free drug, encapsulated drug, particle size, release, lipid composition, and stability are central. For LNPs, RNA integrity, encapsulation efficiency, lipid ratio, ionizable-lipid impurity, potency, and endosomal-escape-related function are key. For albumin-bound products, protein aggregation, particle distribution, reconstitution, free drug, and infusion compatibility are important. For polymeric particles, residual solvent, polymer molecular weight, degradation, burst release, and release kinetics must be controlled [35–41, 68, 69], and these interdependent quality attributes are illustrated in Fig. 3.

**Fig. 3:**
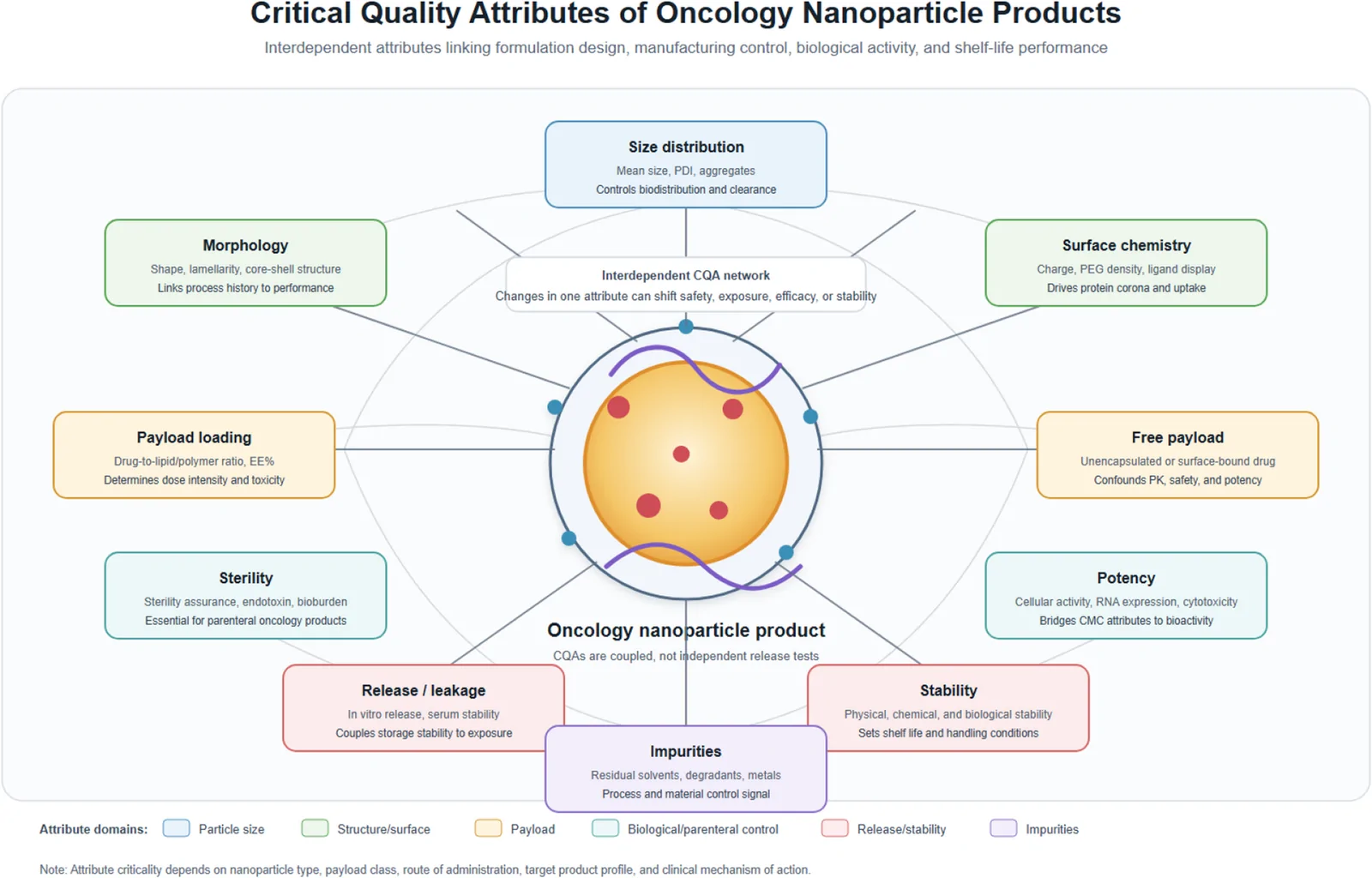
Interdependent critical quality attributes (CQAs) of oncology nanoparticle products. The attributes shown are grouped by the development question each one answers: physical identity (size distribution, morphology, shape), surface and interfacial properties (surface chemistry), payload control (loading, encapsulation), biological performance (cellular activity), and product robustness (sterility, stability, impurities). Arrows indicate that a change in one attribute generally propagates to others, so these attributes cannot be specified independently. Abbreviations: CQA, critical quality attribute. Figure prepared by the authors using Microsoft PowerPoint (Microsoft 365)

A major weakness in many preclinical nanomedicine reports is overreliance on size and zeta potential. DLS measurements are useful but can be biased toward larger particles and may not detect multimodal populations or morphology changes [68]. Orthogonal methods such as nanoparticle tracking analysis, asymmetrical-flow field-flow fractionation, cryo-TEM, electron microscopy, chromatography, capillary methods, mass spectrometry, and functional potency assays should be used when they answer development-relevant questions [68, 69]. Practical examples of relevant CQA domains, why they matter in oncology, and typical analytical tools are summarized in Table 7.

### Functional potency assays for RNA/LNP oncology products

Potency is the attribute most often described as critical and least often described concretely. For an mRNA cancer vaccine the release strategy generally uses a tiered set of assays rather than one test, because no single measurement covers the path from particle to immune response. A workable tier structure is: an identity and integrity tier, a translation tier, and an immunological tier. Representative assays for each tier, and what each one can and cannot establish, are set out in Table 8.

**Table 8:** Representative functional potency and release assays for RNA/LNP oncology products

Tier	Assay	What it measures	Role and limitation
Integrity	Capillary gel electrophoresis or fragment analysis	Percentage intact RNA, fragment profile	Routine release; sensitive and reproducible, but says nothing about delivery or expression [82]
Integrity	Cap analysis (LC-MS or enzymatic assay) and poly(A) tail length	5’ capping efficiency, tail length distribution	Routine release; uncapped RNA both fails to translate and increases innate immune activation [82, 83]
Integrity	RiboGreen or equivalent dye-exclusion assay	Encapsulation efficiency and free RNA	Routine release; a widely used surrogate that is frequently over-interpreted as a potency measure
Translation	Cell-based expression assay in a permissive line (reporter luminescence or antigen-specific immunoassay)	Protein expressed per unit RNA delivered	Primary potency assay; integrates uptake, endosomal escape, and translation, but cell line choice strongly influences the result and must be justified and controlled
Translation	Target knockdown dose-response (siRNA products)	Relative potency against a reference standard	Primary potency assay for gene-silencing products; requires a stable reference standard, which is difficult for individualized products
Immunological	Dendritic cell transfection with T cell co-culture; interferon-gamma ELISpot or intracellular cytokine staining	Antigen presentation and functional T cell activation	Characterization and comparability rather than routine release; donor-dependent, long turnaround, and difficult to qualify to release standards [15]
Immunological	Innate activation panel (cytokine induction in whole blood or PBMC)	Interferon and inflammatory cytokine induction	Safety-relevant characterization; a dose-limiting attribute for RNA payloads [47]

The integrity tier confirms that the RNA is intact and correctly capped, typically by capillary gel electrophoresis or fragment analysis for percentage intact RNA, a cap-specific assay for 5’ capping efficiency, and a poly(A) tail length measurement. These are necessary but not sufficient, because intact RNA inside a poorly performing particle still produces no protein. The translation tier measures expressed protein after transfection of a permissive cell line, usually by reporter luminescence for a surrogate construct or by antigen-specific immunoassay, flow cytometry, or western blot for the actual construct; this is the assay that reports the combined efficiency of uptake, endosomal escape, and translation and is therefore the closest routine readout of what the delivery system actually does. The immunological tier is used for characterization and comparability rather than routine release: dendritic cells are transfected and antigen presentation is confirmed by co-culture with antigen-specific T cells, with interferon-gamma ELISpot or intracellular cytokine staining as the readout, or an ex vivo assay is run using peripheral blood mononuclear cells [15, 83]. For an siRNA product the equivalent translation-tier assay is target messenger RNA or protein knockdown in a relevant cell line with a dose-response curve, from which a relative potency against a reference standard can be calculated.

Two problems limit this area and should be stated plainly. The first is harmonization. There is no compendial potency assay for mRNA products, cell lines and reference standards differ between laboratories, and relative potency values are therefore not comparable across sites or sponsors. Reference materials and consensus procedures are being developed, and the USP draft guidelines on analytical procedures for mRNA vaccine quality are the most advanced public effort, but they remain draft guidance rather than a harmonized standard [82]. The second is specific to individualized products. When each batch encodes a different set of neoantigens, a conventional product-specific reference standard cannot exist, so potency has to be established against a platform surrogate construct with the patient-specific sequence verified separately. That approach is workable but shifts the burden onto demonstrating that the surrogate predicts the behavior of the real construct, which is an open comparability question rather than a solved one.

## Manufacturing, process control, sterility, scale-up, and comparability

Manufacturing is part of nanoparticle product identity. The same nominal composition can produce different particle properties if hydration, mixing, solvent exchange, remote loading, extrusion, homogenization, milling, solvent removal, lyophilization, filtration, or filling conditions change. For LNPs, flow-rate ratio, total flow rate, pH, N/P ratio, lipid concentration, solvent removal, and RNA quality can influence particle properties and potency [13–18]. For liposomes, hydration, sizing, loading gradient, cholesterol content, PEGylation, and sterile processing can influence release and stability [6–8, 36].

It is worth being explicit about which process parameters are critical, because they differ by platform in the same way CQAs do. For microfluidic or impingement-jet LNP manufacture, the critical process parameters are the aqueous-to-organic flow rate ratio, total flow rate, the pH and ionic strength of the aqueous phase, the N/P ratio, lipid concentration, and the rate and completeness of solvent removal; these set particle size, encapsulation, and internal structure, and therefore potency. For liposomes, hydration temperature relative to the lipid phase transition, extrusion pressure and number of passes, the transmembrane gradient used for remote loading, loading time and temperature, and the tangential flow filtration conditions used for buffer exchange are the parameters that move drug-to-lipid ratio and release. For polymeric particles, the organic-to-aqueous ratio, mixing energy, polymer concentration, and solvent removal rate set particle size and burst release. Mapping these parameters to the attributes they control, rather than treating manufacture as a fixed recipe, is what makes a later process change defensible [39, 40].

### Batch-to-batch variability and process analytical technology

Batch-to-batch variability in nanoparticle products comes from three sources that need different controls. Raw material variability is the most underestimated: synthetic lipid lots differ in isomeric purity and oxidation state, cholesterol can carry oxysterols, polymer lots differ in molecular weight distribution and end-group chemistry, and in vitro transcribed RNA differs in capping efficiency and double-stranded RNA content between lots. Process variability arises where an operation is transfer-limited, which for these products is usually the mixing step where supersaturation drives particle formation. Analytical variability is significant because several routine nanoparticle methods, dynamic light scattering in particular, are intensity-weighted and comparatively imprecise for polydisperse samples [68, 69].

Process analytical technology is a better fit for this product class than for most, because the attributes of interest form during the process rather than after it, and an end-of-process size measurement cannot tell the operator why a batch drifted [38, 84]. In-line or at-line measurements that are practical today include in-line dynamic light scattering or focused-beam reflectance during mixing for particle size, in-line ultraviolet and conductivity monitoring across tangential flow filtration to follow buffer exchange and solvent clearance, near-infrared or Raman spectroscopy for lipid and solvent content, in-line pH and conductivity at the point of mixing for LNP formation, and at-line fluorescence dye-exclusion for encapsulation. The objective is not to replace release testing but to establish a design space in which the process is demonstrably under control, which is also what makes later post-approval changes manageable [39–41, 85].

### Scale-up and scale-out

Nanoparticle processes rarely scale by geometric similarity, because particle formation depends on local mixing at a scale much smaller than the vessel. Scaling up a batch mixing step therefore changes the micromixing time and the particle population with it. Two strategies are used. Scale-up increases throughput by making the unit operation larger or by running it continuously, which suits liposomal and polymeric products with conventional batch economics; for LNPs it usually means moving from a single microfluidic chip to a larger-bore impingement jet or a parallelized cartridge while holding the flow rate ratio and Reynolds number in the mixing zone constant. Scale-out replicates the small unit many times in parallel, which is the natural fit for individualized products where each batch is small and the number of batches is the variable. The two strategies have different regulatory consequences: scale-up requires demonstrating that the enlarged process produces a comparable product, while scale-out requires demonstrating that many parallel units produce equivalent product and that the chain of identity between patient, sequence, and batch is never broken.

Scale-up also affects the feasibility of individualized or precision oncology platforms. Personalized mRNA vaccines must be manufactured quickly enough to support a clinically relevant treatment window. That requirement changes the development problem: the product must have a robust sequence-to-batch workflow, reliable analytical release, rapid sterility or microbiological control strategy, and validated chain of identity. For developing-country oncology settings, cost, cold-chain burden, and manufacturing access may be as important as molecular sophistication.

### Sterility strategy

Sterility is a critical translational issue because many oncology nanoparticle products are parenteral. Terminal sterilization may damage particles, alter release, degrade lipids or RNA, or change morphology. Sterile filtration may be difficult for particles near filter pore-size limits, aggregates, high-viscosity dispersions, or delicate vesicles. Therefore, sterile strategy should be designed early rather than treated as a final manufacturing step. Aseptic processing, component sterilization, bioburden control, filter compatibility, container-closure integrity, and stability after filling all need to be integrated into the control strategy [35–41].

### Comparability after manufacturing changes

Manufacturing changes are inevitable across a product lifecycle, and for nanoparticle products they are harder to justify than for small molecules because the product is defined by a distribution of structures rather than by a molecular entity. The framework used for biotechnology products in ICH Q5E transfers well: comparability is demonstrated through a stepwise exercise that begins with analytical comparison and escalates to nonclinical or clinical data only if the analytical package cannot resolve the uncertainty [86]. For a nanomedicine, an adequate analytical comparability package normally needs orthogonal size and distribution methods rather than dynamic light scattering alone, structural confirmation by cryogenic electron microscopy, the full payload-association profile including free drug or free RNA, a discriminatory release or dissolution comparison, the impurity profile including process-related impurities specific to the new process, a functional potency comparison, and accelerated and stressed stability to detect differences that only appear over time [35–41, 68, 69]. Changes that alter the mixing geometry, the lipid or polymer supplier, the solvent removal method, or the sterilization approach carry the highest risk and should be supported by the fullest package. Where a change cannot be shown to be comparable analytically, a bridging clinical pharmacokinetic study is usually the next step, and building that possibility into the development plan early is far cheaper than discovering the need after approval [85].

## Clinical translation and evidence generation

Cancer nanomedicine trials should be designed around the specific problem the nanoparticle is intended to solve. If the objective is toxicity reduction, the trial should capture toxicity differences that matter clinically. If the objective is improved tumor exposure, pharmacokinetic and pharmacodynamic evidence should support that claim. If the objective is intracellular RNA delivery, the development program should include functional readouts such as antigen expression, immune activation, gene knockdown, or relevant biomarker modulation [13–24].

Concrete examples show what this looks like in practice. Where the objective was toxicity reduction, pegylated liposomal doxorubicin was evaluated against conventional doxorubicin with cardiac function as a co-primary concern, and the resulting label is built around that difference rather than around a survival gain [6–8, 61]. Where the objective was solubility and exposure, albumin-bound paclitaxel was compared directly against solvent-based paclitaxel and showed a higher response rate, and its subsequent pancreatic cancer indication rested on a survival benefit in combination with gemcitabine [48–50]. Where the objective was to maintain a drug ratio, fixed-ratio liposomal daunorubicin and cytarabine was tested against the standard 7 + 3 regimen in a defined acute myeloid leukemia population and demonstrated an overall survival benefit there [52]. Where the objective was local radiation enhancement, the hafnium oxide radioenhancer was randomized against radiotherapy alone using pathological complete response as the primary endpoint, which is the endpoint that matches the mechanism [65]. The individualized neoantigen vaccine trial in resected melanoma used recurrence-free survival in an adjuvant population with checkpoint inhibition as the comparator, which matches a mechanism that acts through immune memory rather than through cytotoxicity [19]. In each case the endpoint was chosen to match the claim; the failures summarized in Table 2 generally did not have this alignment.

Patient selection may become increasingly important. Tumor type, vascularity, macrophage content, stromal density, antigen burden, HLA type, immune infiltration, prior treatment, and molecular subtype can all affect response to nanomedicine or RNA-based immunotherapy. Personalized vaccine platforms already depend on tumor and normal sequencing, neoantigen prediction, manufacturing feasibility, and immune monitoring [19–24]. Future trials may need to link delivery-system selection with patient biology rather than assuming one nanoparticle platform will perform equally across all tumors.

It is important to separate biomarkers that are established from those that are exploratory, because this field has a tendency to present the latter as though they were the former. In cancer nanomedicine specifically, no biomarker has been qualified to select patients for a nanoparticle formulation on the basis of delivery. The biomarkers that do have predictive value in trials of these products are the conventional oncology biomarkers that would apply to the free drug as well: HER2 status, hormone receptor status, KRAS mutation status, mismatch-repair status, and similar. Delivery-related measurements such as ferumoxytol-based imaging of tumor macrophage content and nanoparticle deposition are genuinely promising and have shown lesion-level correlation with response to a liposomal product, but they have been evaluated in small pilot studies and are exploratory [63]. Table 9 makes this distinction explicit.

**Table 9:** Biomarkers relevant to cancer nanomedicine, separated by current evidentiary status

Biomarker	Use in cancer nanomedicine	Status	Comment
HER2 status	Patient selection for HER2-targeted liposomal doxorubicin	Validated	Validated for HER2-directed therapy generally, not for nanoparticle delivery; HER2 positivity did not rescue the MM-302 program [45]
Hormone receptor and KRAS status	Standard eligibility and stratification in trials of nanoparticle cytotoxics	Validated	Predicts response to the drug, not to the formulation; applies equally to the conventional comparator [48–50]
Tumor mutational burden and neoantigen load	Feasibility and expected immunogenicity of individualized neoantigen vaccines	Exploratory	Necessary for manufacturability of the product, but a predictive threshold for benefit has not been established [19–21]
Vaccine-induced T cell response (ELISpot, T cell receptor clonality)	Pharmacodynamic confirmation that an mRNA vaccine has worked	Exploratory	Associates with longer recurrence-free survival in early studies and is the most promising candidate for qualification in this class [20, 21]
Ferumoxytol MRI deposition and tumor-associated macrophage content	Lesion-level prediction of nanoparticle deposition and response	Exploratory	Correlated with lesion response to liposomal irinotecan in a pilot study; not replicated at scale and not qualified [63]
PSMA expression	Patient selection for PSMA-targeted polymeric docetaxel	Exploratory	Target expression was present but did not translate into clinical benefit, which is itself an informative negative result [44]
Circulating complement activation markers	Prediction of infusion reactions to pegylated particles	Exploratory	Mechanistically grounded and useful in characterization, but not established as a clinical decision tool [62]

Commercial and access considerations should also be addressed early. A highly complex nanomedicine may be scientifically attractive but clinically irrelevant if it does not improve outcomes, reduce toxicity, enable a new modality, or provide value compared with simpler alternatives. Oncology patients and health systems need products that are not only innovative but also manufacturable, accessible, and clinically meaningful.

## Development-to-translation matrix for cancer nanomedicine

To help compare platforms beyond novelty, this review proposes a qualitative development-to-translation matrix. The matrix is not intended as a validated scoring system. Instead, it provides a practical framework for comparing formulation maturity, analytical maturity, manufacturing maturity, biological rationale, clinical evidence, regulatory readiness, and commercial feasibility, as summarized in Table 11. This structure is useful because many nanomedicine platforms appear promising in early studies but fail to mature across the full development chain.

Because a qualitative matrix of this kind is easy to over-read, three points should be stated before the results. First, the levels are defined explicitly in Table 10 rather than assigned by impression, and each assignment in Table 11 can be traced to those definitions. Second, regulatory maturity and commercial maturity are scored separately, because they are not correlated. A product can be regulatorily mature and commercially unsuccessful, which is exactly what happened to micellar paclitaxel in the European Union, and a platform can have clear regulatory precedent through the device pathway while serving a very small market, which is the position of the intratumoral inorganic products [55, 65]. Third, and most importantly, these are heuristic rankings intended to structure discussion. They are not evidence-based quantitative metrics, they are not derived from a systematic scoring exercise with defined weights, and they should not be used as a substitute for product-specific assessment. Their purpose is to make the basis of a judgment visible so that a reader who disagrees can see precisely where and why.

**Table 10:** Definitions of the maturity levels used in Table 11

Dimension	Low	Medium	High
Formulation and analytical maturity	No consensus manufacturing route; characterization largely research-grade; no orthogonal methods routinely applied	Reproducible manufacture at pilot scale; orthogonal characterization available but not standardized across laboratories	Commercial-scale manufacture demonstrated; orthogonal methods and compendial or guidance-anchored analytical expectations in place
Clinical evidence	Preclinical only, or first-in-human safety data without efficacy signals	Controlled trials completed with mixed results, or benefit shown only in narrow or single-arm settings	At least one randomized trial showing a clinically meaningful benefit that supported approval in a defined population
Regulatory maturity	No approved product in the class; no class-specific guidance; review expectations case-by-case	Approved product exists in at least one region, or class-specific guidance exists but generic and comparability pathways are unsettled	Multiple approvals in major regions, class-specific regulatory guidance published, and comparability expectations established [35–37]
Commercial maturity	No marketed product, or marketing discontinued or withdrawn	Marketed in limited regions or indications; supply or reimbursement constraints significant	Sustained multi-region marketing with established supply, physician adoption, and in some cases generic or follow-on competition

**Table 11:** Proposed development-to-translation matrix for oncology nanoparticle platforms

Platform	Formulation/analytical maturity	Clinical evidence	Regulatory maturity	Commercial maturity	Key next need
Liposomal cytotoxics	High	High for selected products	High	High	Better patient-selection and comparability tools [6–8, 36]
Albumin-bound products	Medium-high	High for selected products	High	Medium-high	Clearer mechanism-to-clinical differentiation [48–53]
LNP/RNA oncology platforms	Medium-high	Emerging	Medium	Low-to-medium	Robust potency, rapid manufacturing, and tumor/immunology biomarkers [13–24]
Polymeric nanoparticles	Medium	Low-to-medium	Low	Low	Clinically relevant release, scale-up, residual-solvent and polymer controls [9, 10, 54]
Polymeric micelles	Medium	Medium for selected products	Medium	Low	Dilution-stability and commercial differentiation [54, 55]
Nanocrystals	High	Low for oncology	Medium	Low for oncology	A defined oncology use case; the platform is mature but answers a solubility question rather than a delivery question [56]
Nanoemulsions, SLNs, and NLCs	Medium	Low	Low	Low	Physical stability on storage and a sterile processing route; oncology-specific clinical data [57]
Inorganic oncology particles	Medium	Medium for local administration	Medium (device pathway in the EU)	Low	Long-term clearance and retention data; evidence that the approach extends beyond accessible lesions [64, 65]
Hybrid/biomimetic particles	Low-to-medium	Early	Low	Low	Reproducible sourcing, consensus characterization and potency standards, sterility, and scalability [1, 2, 35, 59]

Liposomes and albumin-bound products have relatively high clinical and regulatory maturity because approved oncology examples exist. LNP-based RNA oncology platforms have strong technological momentum and growing clinical evidence, but oncology-specific manufacturing, potency, and patient-specific workflow questions remain. Polymeric, micellar, inorganic, and hybrid platforms may offer important scientific opportunities, but many remain less mature commercially and require stronger product-specific evidence before broad clinical adoption. A visual comparison of these relative maturity domains is provided in Fig. 4.

**Fig. 4:**
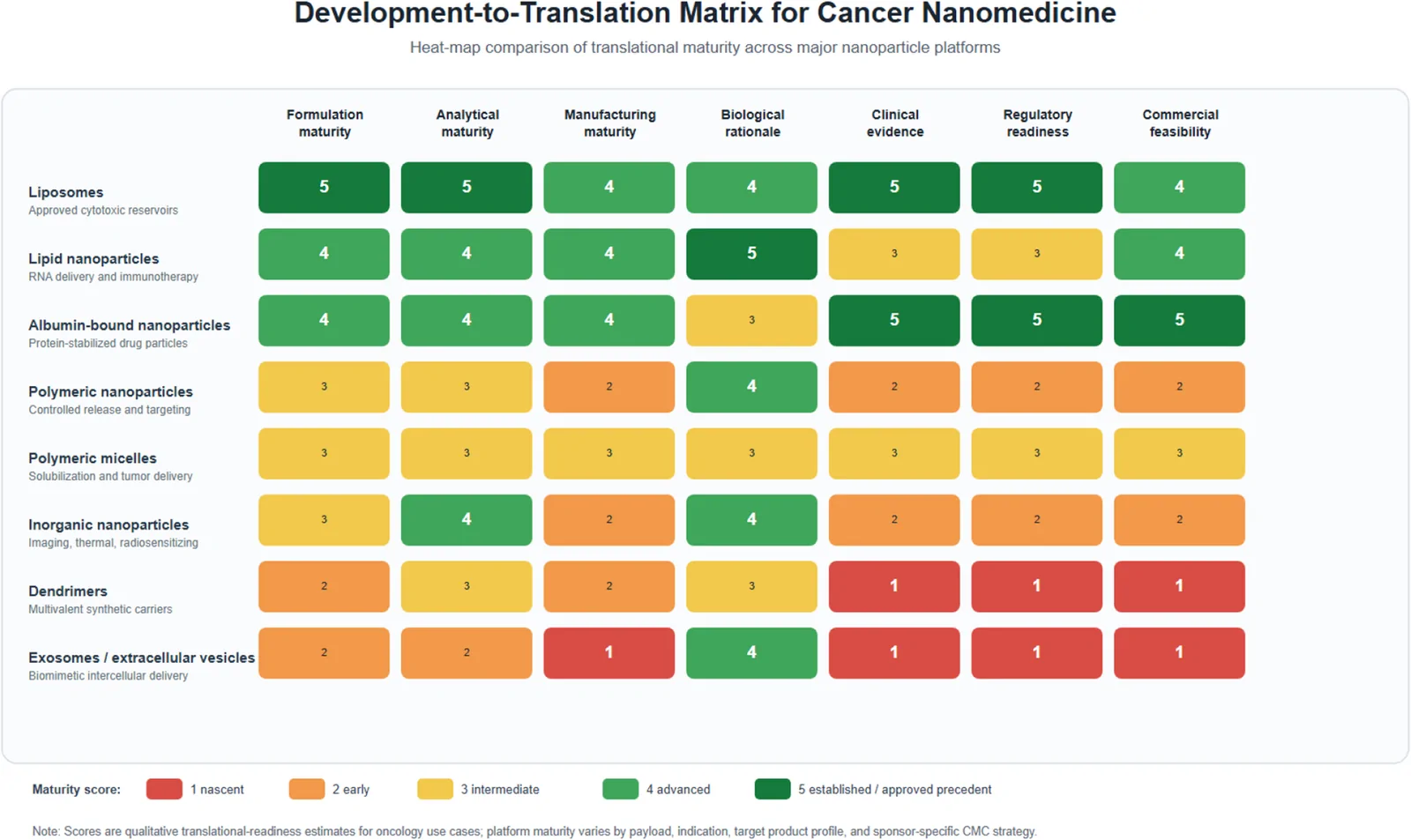
Development-to-translation heat map for cancer nanomedicine platforms. Shading indicates relative maturity across the assessed dimensions, from fully mature platforms such as liposomes to early-stage platforms such as inorganic particles and dendrimers. The levels shown follow the definitions in Table 10 and correspond to the assignments in Table 11. These rankings are heuristic and qualitative; they are intended to structure comparison and are not validated quantitative metrics. Abbreviations: LNP, lipid nanoparticle; RNA, ribonucleic acid. Figure prepared by the authors using Microsoft Excel and Microsoft PowerPoint (Microsoft 365)

## Global access, developing-country oncology, and the platform choice

Cancer nanomedicine is often presented as a high-technology field, but its impact will be limited if products cannot be manufactured, supplied, and used in diverse health systems. This is especially relevant for oncology journals and institutions serving developing countries, where cancer burden is increasing and access to advanced therapies may be constrained by cost, infrastructure, cold-chain requirements, infusion capacity, diagnostic availability, and reimbursement [5].

It is worth being concrete about what those constraints are, because they act at six distinct points and each one favors different platforms. Diagnostics come first: albumin-bound paclitaxel requires only routine histopathology, whereas a HER2-targeted product requires immunohistochemistry with reliable external quality assessment, and a neoantigen vaccine requires tumor and germline sequencing. Sequencing and bioinformatics capacity is the hardest of these to acquire, since an individualized vaccine needs paired tumor-normal whole-exome or transcriptome sequencing, variant calling, human leukocyte antigen typing, and neoantigen prediction turned around fast enough to remain clinically relevant; that infrastructure exists in a small number of centers in most middle-income countries and is often concentrated in one or two academic hospitals. Cold-chain requirements differ by an order of magnitude across platforms: liposomal doxorubicin and albumin-bound paclitaxel are refrigerated or room-temperature products that travel on existing pharmaceutical distribution, while current mRNA/LNP products require frozen or ultra-frozen storage with continuous monitoring [17]. Manufacturing access matters because a liposomal cytotoxic can in principle be produced regionally and generic versions already exist, whereas an individualized vaccine cannot be stockpiled and must be produced per patient, which means either a local manufacturing site or an international shipping window that is often incompatible with the treatment schedule. Regulatory frameworks are the fifth point: nanomedicine-specific guidance and a defined pathway for generic or follow-on complex injectables exist in the United States and European Union but not in most national systems, which slows both innovator approval and generic entry [35–37]. Reimbursement is the sixth, and in practice often the binding constraint, since products priced against high-income markets are unaffordable at the per-patient budgets available in many public oncology systems.

Egypt and the wider Middle East illustrate the point usefully for this readership. Cancer incidence in the region is rising with population growth and aging, liver and breast cancers carry a particularly heavy burden in Egypt, and national registry data have been used to plan services for two decades [87, 88]. The infrastructure picture is uneven rather than uniformly limited: large tertiary and specialist cancer centers in Cairo and several Gulf states have molecular pathology, sequencing, and clinical trial capability comparable to high-income centers, while regional and governorate hospitals treating most patients often do not. Two practical consequences follow. First, individualized RNA products are realistically deliverable in the region only through a small number of hub centers, and any claim that they represent the future of accessible oncology in such settings should be treated with caution. Second, the most valuable near-term contribution of nanomedicine in these systems is likely to come from the least novel platforms: reliable generic liposomal and albumin-bound cytotoxics that reduce severe toxicity, enable outpatient administration, and reduce the supportive-care burden on stretched inpatient services. Regional manufacturing of complex generics is a more plausible route to population-level benefit than technology transfer of personalized products, and the regulatory bottleneck for it is the absence of a settled comparability pathway for complex injectables rather than the science [37, 55, 86].

Platform choice is therefore not separable from access. The manufacturing complexity, cold-chain burden, and cost of a product are determined by the platform selected in early development, usually before anyone has considered where the product will be used. A liposomal or albumin-bound cytotoxic is a low-complexity, refrigerated, regionally manufacturable product whose cost falls once generics enter. A personalized mRNA/LNP vaccine is a high-complexity, ultra-cold, per-patient product whose cost structure does not improve with volume in the same way, because each batch serves one person. Shared-antigen mRNA vaccines sit between the two and are considerably more deliverable than individualized ones for exactly this reason, which is an argument for continuing to develop them alongside personalized approaches rather than treating them as a lesser option [22–24].

Practical innovation should therefore include formulation robustness, room-temperature or refrigerated stability where feasible, simplified administration, clear clinical differentiation, and realistic quality-control requirements. For personalized RNA oncology products, access may depend on whether sequencing, bioinformatics, manufacturing, and release testing can be performed quickly and affordably. For liposomal or albumin-bound cytotoxics, access may depend on reliable supply, generic comparability, and clinical positioning within existing treatment pathways.

From this perspective, oncology nanomedicine should not be judged only by molecular novelty. It should be judged by its ability to improve patient outcomes in real clinical settings. Products that reduce severe toxicity, enable outpatient care, improve tolerability of combinations, or make precision therapy practical can be highly valuable even if the underlying nanoparticle design is less complex than newer experimental systems.

## Future directions

The next phase of oncology nanomedicine is likely to be shaped by four converging trends. The first is integration with precision oncology. Tumor sequencing, neoantigen prediction, immune profiling, and molecular subtype classification will increasingly influence payload selection and patient selection [19–24]. The second is improved delivery biology. Extrahepatic LNPs, tumor-penetrating particles, macrophage-modulating systems, and local delivery strategies may expand the range of addressable tumors if supported by robust evidence [13–18].

The second trend deserves a more specific account of where it currently stands. Extrahepatic LNP delivery has moved from aspiration to demonstrated capability, but only for organs, not for tumors. Selective organ targeting reliably redirects delivery to lung or spleen by altering the adsorbed protein corona, and the mechanism is understood well enough to be designed rather than screened [76, 77]. Polymer and lipopolyplex carriers have achieved route-dependent organ selectivity and lymph node delivery without any targeting ligand [73–75]. Tumor-penetrating designs and macrophage-modulating systems remain largely preclinical. The honest summary is that the field has learned to steer nanoparticles between major organs and has not yet learned to get a useful fraction of an intravenous dose into a solid tumor, and that the near-term clinical gains from this work will come through lymphoid and immune targeting rather than through direct tumor delivery.

The third trend is computational and AI-assisted design. Machine learning may help prioritize ionizable lipids, predict formulation behavior, interpret multiparameter characterization data, or optimize neoantigen selection. However, computational predictions will need experimental confirmation, clinically relevant potency assays, and validated manufacturing controls. The fourth trend is lifecycle product discipline. Nanoparticle products are complex systems; post-approval changes, generic development, comparability, and supply reliability require deep understanding of process-product relationships [35–41].

On the third trend, the state of the art is more advanced than it was two years ago and its limitations are correspondingly clearer. Deep learning models trained on combinatorial ionizable-lipid libraries can now prioritize candidates that outperform benchmark lipids in vivo, and machine learning combined with high-throughput synthesis has produced lipids with organ-selective behavior that were not obvious from medicinal chemistry intuition [89–91]. These are real advances. They are also constrained in ways that matter for anyone planning to rely on them. Training data are heavily biased toward hepatic delivery of a small number of payloads in a small number of mouse strains, because that is where the published data are, so model performance degrades exactly where the clinical need is greatest, which is extrahepatic and tumor delivery. Negative results are rarely published, so the datasets are additionally biased toward compositions that worked. Assay and laboratory differences mean that data aggregated from multiple sources are not strictly comparable, and the field lacks the standardized reporting that would fix this. Most models predict a single in vitro or biodistribution endpoint rather than a therapeutic outcome, and a lipid optimized for transfection efficiency has not been optimized for tolerability, stability, impurity profile, or manufacturability. Every computational prediction therefore still requires wet-lab confirmation, followed by the same formulation development, potency qualification, and manufacturing work as any other candidate. Machine learning is at present a way to search a chemical space faster, not a way to shorten the development path that follows.

For cancer nanomedicine to progress, development programs should avoid overclaiming passive or active targeting without evidence. They should define the intended biological compartment, prove functional delivery, use orthogonal characterization, build discriminatory release and potency methods, and demonstrate clinical advantage. The most successful platforms will likely be those that integrate formulation science, tumor biology, immunology, manufacturability, and patient-centered clinical strategy.

## Conclusions

Cancer nanomedicine has evolved from a formulation strategy for modifying cytotoxic-drug exposure into a broader oncology platform for controlled delivery, RNA therapeutics, immune modulation, and precision therapy. Clinically established products such as liposomal doxorubicin, albumin-bound paclitaxel, liposomal irinotecan, liposomal daunorubicin/cytarabine, and albumin-bound sirolimus particles show that nanoparticle design can create meaningful clinical products when it solves a defined therapeutic or pharmaceutical problem.

The field now faces a more demanding translation standard. Tumor heterogeneity, variable EPR effects, mononuclear phagocyte clearance, protein-corona formation, limited tissue penetration, and inefficient endosomal escape can all limit clinical benefit. At the same time, development issues such as particle characterization, release testing, potency, sterility, scale-up, stability, and commercial feasibility determine whether a promising concept can become a usable medicine.

Two clarifications follow from the comparisons made in this review. The delivery problem is sequential rather than uniform, and platforms should be selected and criticized on the specific step at which they are rate-limiting rather than on generic targeting claims. And the candidates that failed in the clinic after promising preclinical accumulation are as informative as the products that succeeded, because they show consistently that interspecies pharmacokinetic scaling is unreliable, that the relevant comparator is the conventional formulation of the same drug, and that biological activity has to be demonstrated in patients rather than inferred from particle properties.

Future progress will depend less on making nanoparticles more complex and more on making them more clinically purposeful. A successful oncology nanoparticle product should have a clear QTPP, justified platform selection, controlled CQAs, scalable manufacturing, meaningful potency assays, evidence-linked release testing, and a clinical strategy that identifies the patients most likely to benefit. Under this integrated development model, nanomedicine can remain an important contributor to precision oncology rather than a field defined only by material novelty.

## Data Availability

No datasets were generated or analysed during the current study.
